# A-Site Cation Dependence of Self-Healing in Polycrystalline APbI_3_ Perovskite Films

**DOI:** 10.1021/acsenergylett.3c00017

**Published:** 2023-05-03

**Authors:** Pallavi Singh, Yahel Soffer, Davide Raffaele Ceratti, Michael Elbaum, Dan Oron, Gary Hodes, David Cahen

**Affiliations:** † Dept. of Molecular Chemistry and Materials Science, Weizmann Institute of Science, Rehovot 76100, Israel; ‡ Dept. of Physics of Complex Systems, Weizmann Institute of Science, Rehovot 76100, Israel; § CNRS UMR 9006-IPVF Institut Photovoltaïque d’Ile-de-France, 18 Boulevard Thomas Gobert, Palaiseau 91120, France; ∥ Dept. of Chemical & Biological Physics, Weizmann Institute of Science, Rehovot 76100, Israel

## Abstract

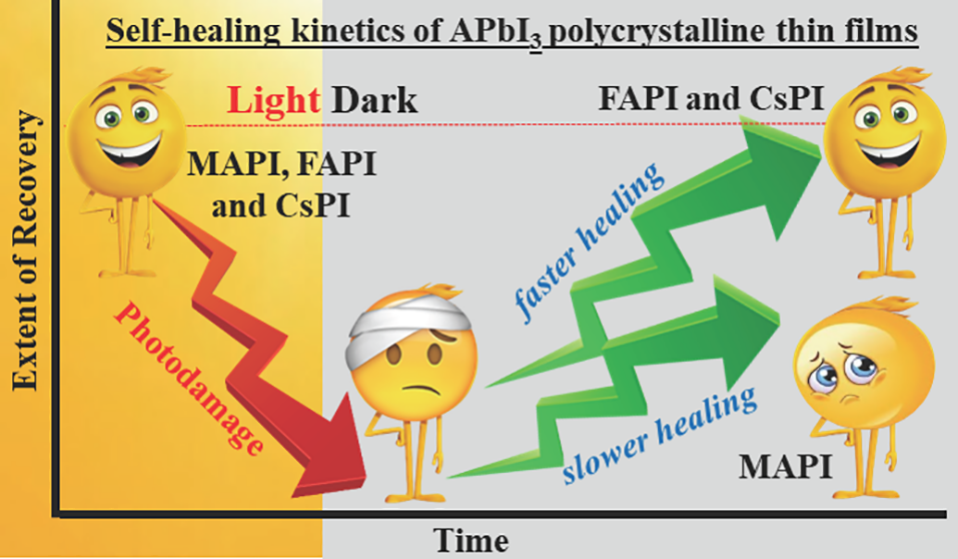

In terms of sustainable use, halide perovskite (HaP) semiconductors have a strong advantage over most other classes of materials for (opto)electronics, as they can self-heal (SH) from photodamage. While there is considerable literature on SH in devices, where it may not be clear exactly where damage and SH occur, there is much less on the HaP material itself. Here we perform “fluorescence recovery after photobleaching” (FRAP) measurements to study SH on polycrystalline thin films for which encapsulation is critical to achieving complete and fast self-healing. We compare SH in three photoactive APbI_3_ perovskite films by varying the A-site cation ranging from (relatively) small inorganic Cs through medium-sized MA to large FA (the last two are organic cations). While the A cation is often considered electronically relatively inactive, it significantly affects both SH kinetics and the threshold for photodamage. The SH kinetics are markedly faster for γ-CsPbI_3_ and α-FAPbI_3_ than for MAPbI_3_. Furthermore, γ-CsPbI_3_ exhibits an intricate interplay between photoinduced darkening and brightening. We suggest possible explanations for the observed differences in SH behavior. This study’s results are essential for identifying absorber materials that can regain intrinsic, insolation-induced photodamage-linked efficiency loss during its rest cycles, thus enabling applications such as autonomously sustainable electronics.

The APbX_3_ Pb halide perovskites (HaPs), with A being a monovalent cation and X a halide anion, are attractive materials for inexpensive yet very efficient thin polycrystalline film solar cells,^1,2^ light-emitting diodes,^3,4^ and radiation^5,6^ and particle detectors.^7^ However, doubts about the stability of the devices and even of the materials themselves overshadows their outstanding performance because, under certain conditions, HaPs degrade during exposure to intense radiation and humid conditions.^8,9^ To date, most studies on stability have been done on complete devices with a multicomponent architecture, where it is likely that changes occur at interfaces and in the non-HaP parts of the devices.^10^ Therefore, it is challenging to extract the extent and kinetics of HaP material degradation by itself. Such is also the case when self-healing (SH) in the absorber material is proposed to explain device recovery from damage.^11−15^

Earlier, we studied the recovery from photodamage in unencapsulated, high-bandgap bromide-^16,17^ and iodide-based^18,19^ HaP single crystals by confocal fluorescence microscopy. We achieved this by using sub-bandgap two-photon (2P) confocal illumination, which allows excitation well into the crystal interior. Thus, we could follow changes in band-to-band photoluminescence (PL) as a reporter for recovery of damage inflicted by such excitation. We also studied recovery kinetics near and at the surface of such crystals, damaged with direct above-bandgap excitation, using one-photon (1P) confocal microscopy.^17^ The latter study was done without (transparent) encapsulation, so reactions with the ambient environment and the escape of volatile degradation products were possible. Such an approach for SH is not well-suited for the air-sensitive thin, polycrystalline films that lie at the heart of photovoltaic and LED devices.

Therefore, we now report on the SH kinetics of encapsulated Pb iodide-based polycrystalline thin films using photoluminescence (PL), i.e., fluorescence recovery after photobleaching (FRAP) with supra-bandgap (i.e., 1P) excitation. Being able to perform such an experiment significantly broadens the scope of studying SH after photodamage via PL kinetics, which hitherto relied on single crystals.^17^ Notably, SH of mechanical damage due to externally applied mechanical stress was studied first with polycrystalline films^20^ and then with single crystals.^21^

We compare three APbI_3_ iodide perovskites to explore how the nature of the A cation, from the relatively small inorganic Cs through medium-sized MA to larger FA (where the last two are organic cations), affects PL recovery. The three APbI_3_ compounds are the room-temperature tetragonal β-phase of MAPbI_3_ (referred to as MAPI), the high-temperature (HT) photoactive either cubic or trigonal α-FAPbI_3_ (FAPI),^22,23^ and the low-temperature (LT) photoactive orthorhombic γ-CsPbI_3_ (CsPI) phases.^24^ We chose iodide-based Pb perovskites because their electronic transport properties generally surpass those of the bromides or chlorides. Also, their absorption thresholds are close to the optimum for maximum solar photovoltaic (PV) conversion efficiency, explaining the strong interest in using them in PV cells. At the same time, though, iodides are the least stable Pb halide perovskites; thus, SH is even more critical for them than for other HaPs.

The encapsulation (Scheme 1, detailed in section 1.3 in the Supporting Information) used in this study not only protects the films from reacting with moisture and O_2_ from the ambient environment but also assists in self-healing by restricting the escape of gaseous degradation products from the film, thus facilitating re-formation of the original material. Here, we used PIB (poly isobutylene), a gas-impermeable rubber, with very low O_2_ and H_2_O-vapor transmission rate and has been widely used in HaP encapsulation for outdoor stability studies.^25,26^ We preferred using the nonpolar aliphatic PIB polymer instead of other widely used polar polymers such as PMMA, PVA, and PEG. The reason is that the latter are not only known to passivate the HaP surfaces but also tend to absorb/react/interact with the HaP photodamage products. In addition, PIB also does not interact electronically with the HaP. By studying the SH behavior of the thin films by photon excitation through the perovskite–glass interface (see Scheme 1b) any possible effect of polymer/HaP interactions is further minimized.

**Scheme 1:**
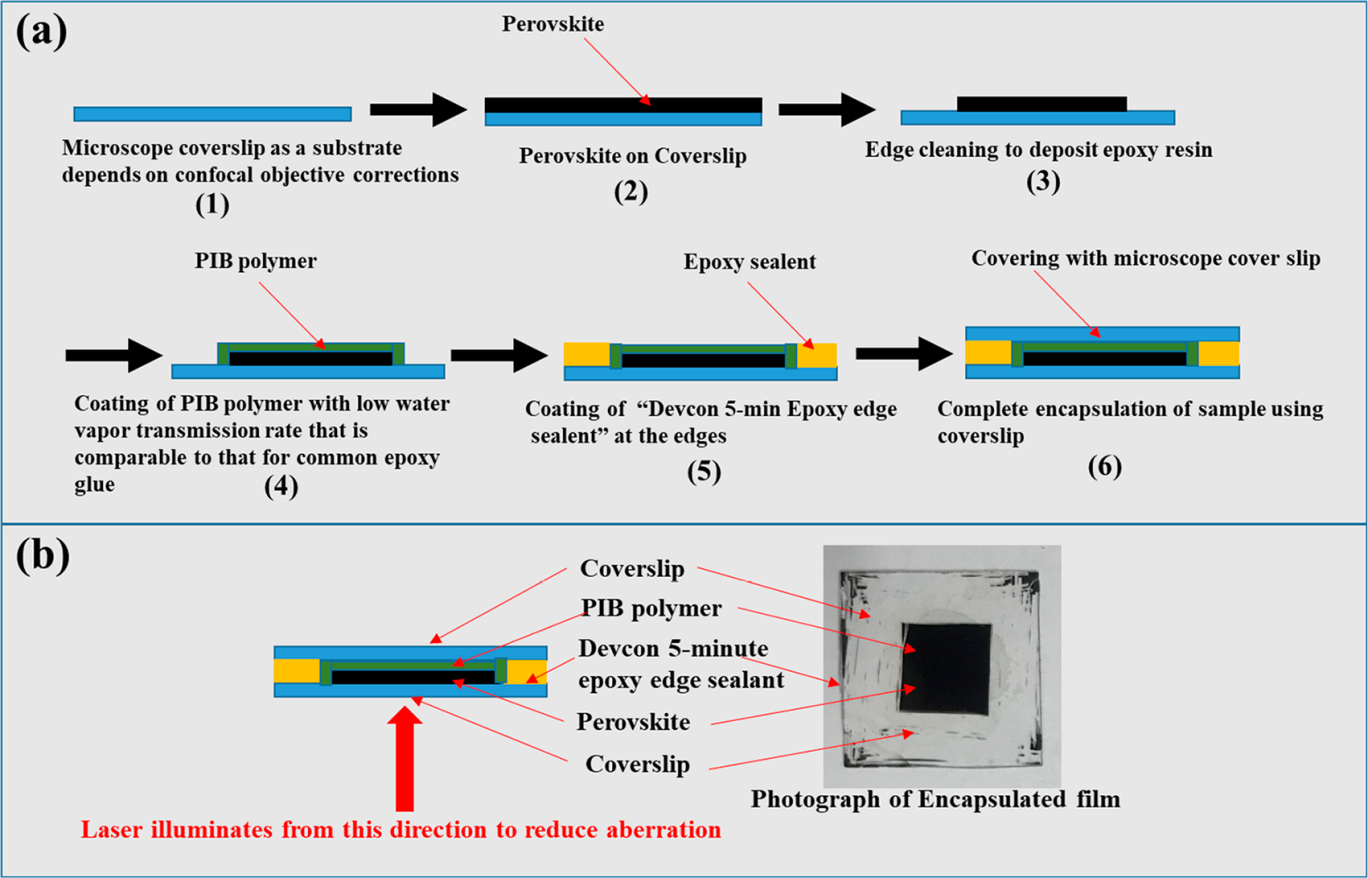
(a) Encapsulation Scheme of Polycrystalline Thin HaP film for Self-Healing Measurements Using FRAP and (b) (right) Photograph of Encapsulated Sample and (left) Illumination Geometry

PIB-encapsulated MAPI films could be kept outside in an ambient environment for more than one year (did not change color). Encapsulated FAPI films (normally stable at >150 °C) could be kept in an ambient environment for more than half a year without color change. In an ambient environment unencapsulated CsPI turns yellow within 1 h, but with encapsulation it does so only after several days. Thus, this encapsulation allows us to compare the SH of black photoactive phases even at room temperature. We note that for unencapsulated samples in air the phase transformation from the black photoactive to the yellow photoinactive phase occurs within short time intervals.^27^Figure S1 shows the transition from a photoactive to a photoinactive phase, which is 2–3 days for FAPI_3_ and 1h for CsPI_3_ in our case. Thus, a very significant finding of this study is that by encapsulating the HaP films, these compositions show sufficient self-healing capabilities to render them stable during hours of nonconcentrated sunlight exposure, with recovery during night time or operation as an LED; possibly, this feature enables their use as radiation/particle beam detector materials.

Before the photodamage and healing experiment, the polycrystalline thin films were characterized by powder X-ray diffraction to confirm phase purity. The detailed XRD measurement results appear in Figures S3–S5 in the Supporting Information. The films were between 0.3 and 0.5 μm thick, which means that they absorb well above 90% of the incident supra-bandgap illumination.

## Comparison of Photodamage Thresholds

We compare, via PL, the FRAP response of encapsulated polycrystalline thin films of MAPI, FAPI, and CsPI to intense laser illumination, using 1P confocal microscopy. The method is detailed in section 3 in the Supporting Information and summarized below.

The sample is illuminated with a supra-bandgap continuous laser (488 nm or, where expressly noted, 405 nm), and the optimal laser power for imaging via PL is determined. After obtaining this reference image, the sample is, within a few seconds, exposed to laser powers that are up to 50–70× higher than that used for the imaging. The laser power is increased until a significant (few %) change from the initial PL intensity is seen, a change interpreted as “damage”. In this way, localized photodamage is inflicted on several regions of interest (ROIs, cf. Figure 2) of the thin film from its surface inward. In this part of the experiments, we use a preprogrammed pattern of a combination of rectangular and circular ROIs (Figure S2 and Figure 2) where each of these is exposed to a different laser power; the corresponding power densities are given in Table S1. Subsequently, the change in PL intensity toward its value before damage is monitored over time as a proxy for SH.

**Figure 1:**
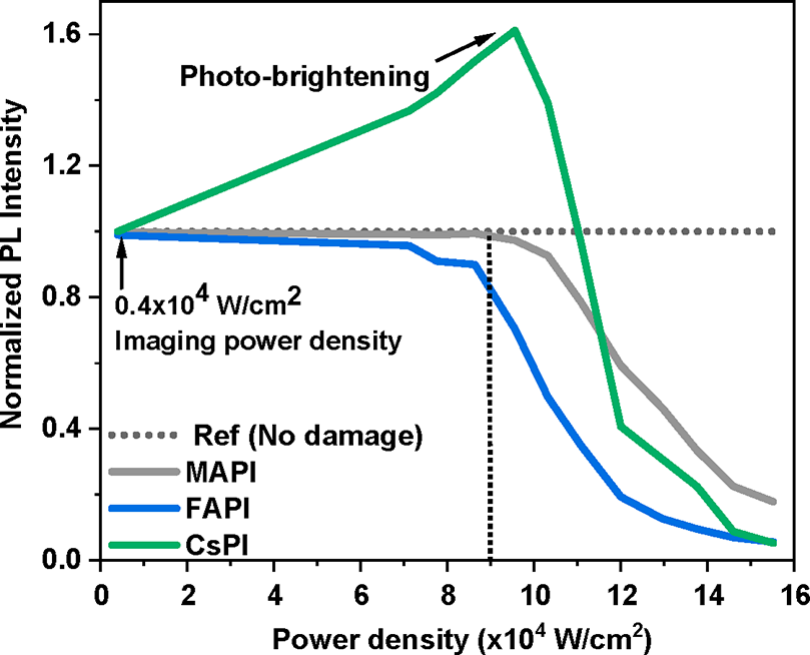
Photoluminescence (PL) intensity as a function of 488 nm laser power densities used to cause photodamage (measured as change in PL intensity), immediately (∼3 s) after photodamage. The PL intensity is normalized to that before photodamage. The baseline PL signal (before photodamage; horizontal dotted line) and any further PL imaging were obtained with 0.4 × 10^4^ W/cm^2^ power density for all three materials. The maximum power density of 1.55 × 10^5^ W/cm^2^ used here gave ∼80–90% PL loss for all three materials. The vertical dotted line shows the MAPI damage threshold to which those of the other two materials are compared (in the text). The data are averaged over five experiments per sample for two samples.

For the 488 nm laser the pixel dwell time is 7.2 μs, corresponding to a deposited energy density of ∼0.5 J/cm^2^ at the threshold (section 5 in the Supporting Information). The absorbed energy density corresponds to several tens of seconds of sunlight. The power density at the damage threshold is equivalent to about 1 million suns (applied for ∼7 μs). This strong, short illumination creates new chemical species (i.e., defects) of the same types as those created by very weak continuous illumination, consistent with the results shown in Figure 1. That figure shows a roughly linear dependence of PL loss (i.e., defect formation) on increase in laser power density for FAPI and MAPI (the more complex behavior of CsPI is discussed later). Such a dependence implies that the inflicted photodamage correlates with the number of defects created by the laser, according to its power density, beyond the damage threshold. Exceptions arise when illumination increases the temperature to allow overcoming thermal activation energies for irreversible processes or excites charges to densities that induce Auger recombination. Such conditions are not reached in our process (see section 4 in the Supporting Information for the calculation of the temperature increase (≤1 °C)).

The dependence of the extent of photodamage on the illumination power (i.e., photodamage threshold) for the three studied materials is presented in Figure 1. Here the photodamage threshold corresponds to the laser intensity that created enough defects to be still observed after the end of the bleaching procedure of the whole ROI (it takes 3 s to complete an illumination cycle). We kept the exposure time constant and studied the effect of laser power density on the extent of degradation for each sample.

Comparing photodamage thresholds, we find small differences between the materials: for MAPI, damage starts from an illumination power density of ∼1.0 × 10^5^ W/cm^2^, for FAPI already at ∼0.8 × 10^5^ W/cm^2^, and for CsPI only from 1.1 × 10^5^ W/cm^2^, a power density at which MAPI already shows 10–30% and FAPI 40–80% PL loss. However, since photobrightening occurs in CsPI and is followed immediately by a steep drop in PL intensity, the damage threshold is probably at lower laser intensity than that at which the PL peaks. The similar photodamage thresholds for CsPI and MAPI cannot be due to local transient heating because the thermal stability of CsPI is much higher than that of MAPI. We also checked the uniformity of the photodamage threshold by measuring at several spots on the same sample and on different samples of the same material. While the distribution of the extent of photodamage at a given power density is very narrow for MAPI and CsPI, it is somewhat larger for FAPI (Figure S6).

AFM experiments to look for pit formation by laser ablation on the MAPI samples showed no pits up to much higher laser intensities, where PL loss was 90% or more (section 7, Scheme S1 showing the different encapsulation used for the AFM measurements, and Figure S7 in the Supporting Information). The pit formation at very high laser intensities implies complete structural damage, while we assume that at lower damage levels, the main damage is caused by at least local ion displacement (not limited to halide ions but including MA and possibly even Pb ions).

**Figure 2:**
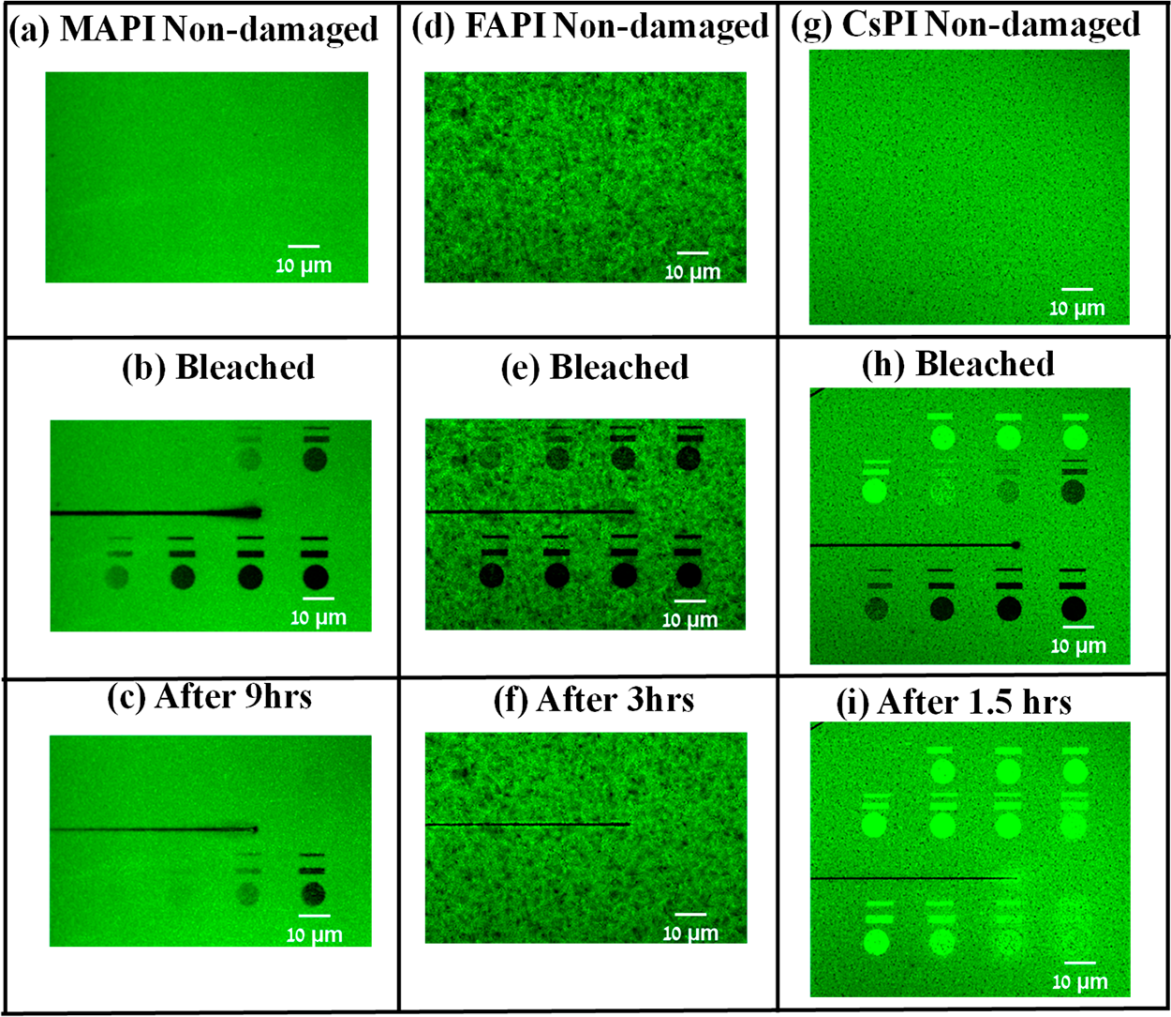
PL images of recovery from photodamage on encapsulated polycrystalline films of MAPI (left column), FAPI (middle column), and CsPI (right column); the experimental conditions are described in section 3 in the Supporting Information. The incident laser power density increases from left to right and from top to bottom. The three images in the top row show confocal images of the films (a, d, and g) before photodamage, i.e., at *t* < 0. The middle row contains images (b, e, and h) just after photodamage, i.e., at *t* = 0. The last row (c, f, and i) shows the PL images of the healed surfaces after 9 h for MAPI (c), 3 h for FAPI (g), and 1.5 h for CsPI (k). In (b), the top ROI shows anomalously greater damage than the subsequent ROI at the lower left; this is likely due to heterogeneities in the film (at either of these two ROIs). In any case, also the anomalous top right ROI is healed completely after 9 h. The black horizontal lines shown in (b), (c), (e), (f), (h), (i) are due to an artifact in the programmed pattern used for damaging but, as can be seen, did not influence the measurements of the ROIs.

**Figure 3:**
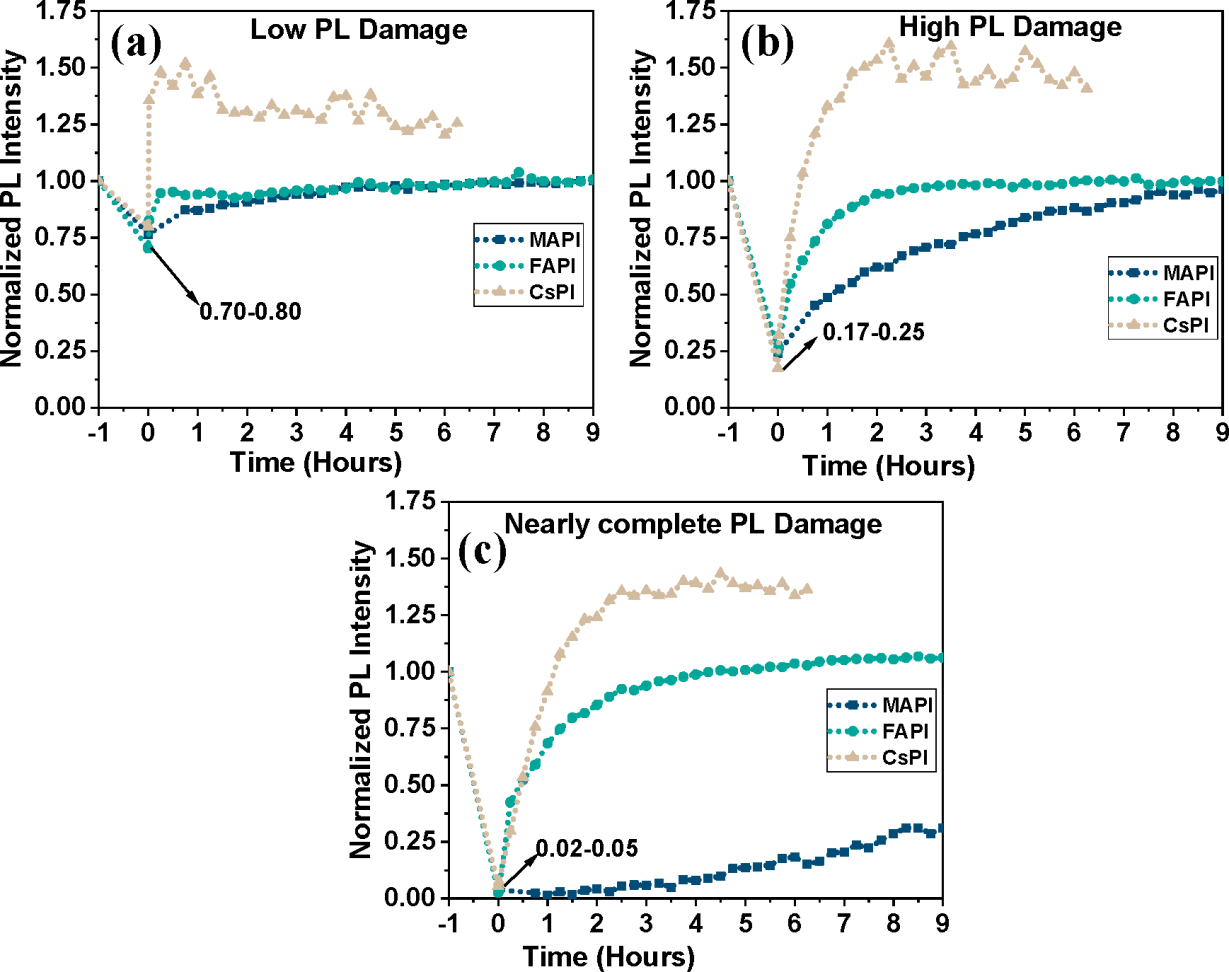
The self-healing kinetics of several damaged ROIs for each of the three types of films for three different degrees of photodamage, as indicated in the figure legends. PL intensities were normalized to that of the adjacent, undamaged area. Each data point shows the average PL intensity of a given ROI at the time, indicated on the *x* axis. PL signals were collected at wavelengths >700 nm. Numbers indicated by arrows in each figure represent the fraction of remaining PL at ROIs at *t* = 0 after exposure to the different laser power densities: low, ∼0.95 × 10^5^ W/cm^2^ (FA) and ∼1.2 × 10^5^ W/cm^2^ (MA, Cs); high, ∼1.2 × 10^5^ W/cm^2^ (FA), 1.3 × 10^5^ W/cm^2^ (Cs), and 1.4 × 10^5^ W/cm^2^ (MA); nearly complete, 1.55 × 10^5^ W/cm^2^. The time difference between *t* < 0 and *t* = 0 is about 3 s, i.e., it took ∼3 s after photodamage to record the first imaging PL (@ 0.04 × 10^5^ W/cm^2^); after that, healing was recorded at intervals of 15 min.

The photobrightening that occurs in CsPI following irradiation (Figures 1–3) does not change the position of the PL spectrum, thus indicating the retention of an intact γ-CsPI phase (Figure S8a). This photobrightening in the material lasts at least several months as long as the samples are not exposed to ambient air. γ-CsPI nanocrystal films were previously found to undergo reversible photobrightening if exposed to light in ambient air. This effect was attributed to surface states caused by humidity, passivated by illumination; apparently, this photobrightening was not reversible under an inert atmosphere.^28^

Fluorescence lifetime imaging on the brightened areas, using a ps pulsed laser, showed lifetimes in the ns range (Figure S8b). The fluorescence lifetime of the nonbrightened regions was ∼0.6 ns (average of 4 spots), whereas, for brightened areas, it was ∼0.9 ns (average of 5 spots), depending on the power density. This increase in the lifetime of emission from a photobrightened area is consistent with a reduction in nonradiative recombination.^28,29^

While for CsPI photobrightening occurred within the exposed ROIs, in some but not all FAPI films, we found PL enhancement at the periphery of ROIs, at higher power densities ((1.1–1.5) × 10^5^ W/cm^2^; Figure S9 gives an example for photobrightening in a FAPI film). This finding indicates that photodamage to, and SH dynamics in, the film has both a temporal and a spatial component. We will analyze and discuss this phenomenon in a future publication.

## Possible Origins of Different Photodamage Thresholds

The fact that FAPI has a lower damage threshold than MAPI might seem surprising since, apart from phase change issues, FAPI is usually considered to be more stable. A possible reason for the reduced photostability of FAPI is that FA^+^ is bulkier than MA^+^ or Cs^+^, which leads to increased tilting of the PbI_6_ octahedra and lattice distortion.^30,31^ As noted, we work with a metastable phase of FAPbI_3_ (the photoactive α-phase); δ-FAPbI_3_, which is photoinactive, is the stable phase at room temperature. One can surmise that light will create defects more readily in a metastable phase than in a thermodynamically stable phase, assuming no significant differences in kinetic barriers to defect formation.

## Comparison of Self-Healing Kinetics

We consider ROIs of the three types of films that show, within each type, similar changes from the predamage PL intensity (low, high, and near-complete damage). We then compare their PL recovery as a function of time (Figure 2) over up to 9 h (Figure 3). To check the reproducibility of PL recovery kinetics, we have performed the measurement in two different setups, using different approaches to photodamage and the measurement of their recovery. The results (see Figures S10–S12) show that PL recovery/SH kinetics are similar in the two setups: i.e., similar to that shown in Figure 2.

Notably, both FAPbI_3_ and CsPbI_3_ form thermodynamically stable, yellow, photoinactive δ-phases at RT. To confirm the re-formation of the original, thermodynamically less stable, photoactive phases, we collected the PL emission spectra during the entire time period (Figures S13–S15; the spectra for MAPI are given, for comparison, in Figure S13). No change in the spectra was observed, indicating that re-formation after damage leads to the same phases as the initially prepared and photodamaged phases.

Results of the SH experiments on all three materials are shown in Figures 2 and 3. In Figure 2, the first (Figures 2a–c), second (Figures 2d–f), and third (Figures 2g–i) columns of the microscope images show SH results on MAPI, FAPI, and CsPI, respectively. In each column, the topmost image shows the PL image of the undamaged film. The second image shows the PL immediately after photodamage (where each of the ROIs was illuminated at an excitation power that increased from left to right and top to bottom). The third image is the PL image after recovery. Figure 3 presents agglomerated data in graphical form on the emission recovery dynamics from circular ROIs within the images, showing a comparison of the SH kinetics for the three different materials for three different degrees of damage.

The previously noted photobrightening of CsPI is clear from Figure 2h,i, as well as from Figure 3. This photobrightening means that in Figure 3, where intensities are normalized to those before damage, there are data for CsPI above the normalized value of 1, which is not the case for MAPI and FAPI.

With this in mind, we can see that CsPI and FAPI initially exhibit similar SH kinetics. At the same time, FAPI also shows a small component of slower SH at longer times in the case of nearly complete damage (Figure 3c). The kinetics, in that case, have an apparent biexponential shape (fast initial increase and much slower later increase), which suggests two different SH mechanisms. The most dominant feature, however, is the striking difference between SH kinetics in FAPI and CsPI compared to that of MAPI. MAPI heals much more slowly and, at nearly complete damage, does not even fully recover after over 9 h (Figure 3c). A secondary effect is that after almost complete photodamage, initially, FAPI recovers even faster than CsPI. Still, the roles reverse after the first half hour (Figure 3c).

The obtained trend of healing kinetics in this study can be compared with that found in a previous 1P study^17^ on and near the (ambient-exposed) surfaces of Pb bromide perovskite single crystals using 488 nm laser excitation. In that study, the extent of healing 12 h after very strong photodamage was CsPbBr_3_ > FAPbBr_3_ > MAPbBr_3_ (Figure 1 in ref (17)). Near the surface of single crystals, only CsPbBr_3_ nearly completely recovered, whereas FAPbBr_3_ recovered partially and MAPbBr_3_ only weakly. We can understand these differences by considering that the single-crystal surfaces were not encapsulated; thus, volatile degradation products (especially from MAPbBr_3_) could escape, and surface reactions with the ambient environment could occur. However, in the present case of encapsulated films, FAPI and CsPI show complete healing even from spots that lost nearly 95–98% of their PL.

Notably, for CsPbBr_3_ crystals, which exhibited near-complete healing at/near the crystal surface, a blue shift of the PL occurs (Figure 2a in ref (16)). However, for the encapsulated films studied here, no significant change in the PL emission spectrum was observed (Figure S8a), as is also the case for healed volumes inside MAPbBr_3_ single crystals.^17^

To compare the kinetics of the SH of the iodide perovskites with the bromide perovskites discussed above, we plotted the bromide data from ref (17) in the same form as that used in the present report, i.e., the normalized intensity of the PL vs time for different extents of initial damage (Figure S16). In general the bromides SH faster than the iodides, but it is clear that MA shows the slowest SH behavior for both halides. While for the bromides, Cs SH is much faster than that of FA, these two cations show similar overall rates for the iodides (within the complication of the CsPbI_3_ photobrightening).

We can also compare the iodide SH rates with those occurring inside the bulk, rather than at and near the surface, of these bromide single crystals, by measuring with two-photon (2P) excitation^16,17^ as shown in Figure S17. In the case of 2P experiments on the bromides we do not have enough data to cover all the different extents of damage, used in the present report. Therefore, for the 2P experiments we show the SH rates of the three APbBr_3_ crystals in two sets of plots—one showing SH over a short time and the other, for the same experiments, over a longer time. In this way the plots convey the results, notwithstanding the very large difference in SH rates between the CsPbBr_3_ crystals and the other two APbBr_3_ crystals. We note that we cannot measure rates for MAPbBr_3_ for medium or highly damaged samples, because of the strong photobrightening that occurs under these conditions. However, it is clear that in contrast to the iodide results and the 1P results on the bromides, SH inside FAPbBr_3_ is much faster than that inside CsPbBr_3_. The difference between the surface and bulk measurements on the bromides for these two cations can be due to surface oxidation of Cs at high laser intensities or by loss of FA in the 1P measurements, as the crystals were not encapsulated.

Further, slightly changing the composition of halide perovskites can substantially change their optoelectronic properties. This is because these have a nonlinear dependence on the defect density in the material and the composition/chemical potential of the relevant chemical species (for example, I_2_). This is not the case for the reactions of degradation and self-healing, which, to a first approximation, will depend linearly on the concentrations of the involved species. In particular, self-healing involves re-establishing a system’s equilibrium that was disturbed by the strong illumination. These reactions, and specifically their kinetic constants, are independent of the optoelectronic state of the perovskite. Therefore, the information obtained in this report applies also to samples that do not have the same initial/equilibrium defect density (and optoelectronic properties), as can be the case if samples are prepared by different researchers (derived in section 8 in the Supporting Information).

## Origin of Differences in Self-Healing Kinetics

Below we suggest several possible causes for the effect of the A cation on SH kinetics. However, there is likely more than one single cause involved in these kinetics. Also, the different causes are not necessarily independent; they are often interrelated.

Self-healing in lead halide perovskites is likely connected to the shallow energetic landscape of the material and the strong dynamic nature of the (Pb–X) matrix,^32,33^ which is also expressed by significant anharmonicity of the lattice vibrations.^34^ Among possible thin films, chemical causes for the differences in SH kinetics between the three HaPs we studied here; a likely one is differences in lattice distortion due to the different A cation size relative to the Pb–I sublattice.^30,31^ The tolerance factors (TFs) of FAPI (TF = 1.0) and CsPI (TF = 0.81) lie at the two outer borders of the range in which the ideal perovskite structure forms, with MAPI in between (TF = 0.89).^35^ Our results show that the HaPs with distorted perovskite matrices, CsPI and FAPI,^30,36^ heal more quickly than MAPI with a less distorted matrix.

Another possible cause could be the difference in hydrogen bonding among the three HaPs. CsPI has no H-bonding capability. While FAPI can form up to four H-bonds compared to MAPI’s three, the results of recent computational theory were that MAPI exhibits stronger overall H-bonding than FAPI, possibly because of the larger dipole on MA^+^ than that on FA^+^.^37^ This expected order of H-bonding strength (MA > FA > Cs) anticorrelates with what we find for SH kinetics. Thus, *if* H-bonding strength would dominate the ability of SH, such anticorrelation would imply that the weaker the H-bonding, the faster the SH.

The possibility that differences in A cation diffusion play a role might seem low, given that the low diffusion coefficients deduced (from NMR experiments) for MA^+^ in MAPI are <10^–16^ cm^2^/s.^38^ While we have not found values for Cs^+^ or FA^+^ diffusion coefficients in the literature, in a theoretical study, Pazoki et al. calculated migration energy barriers for the A cations in MAPI, FAPI, and CsPI and found they decreased in the order MA > FA ≈ Cs.^39^ This could explain our results (that FAPI and CsPI heal much more quickly than MAPI), if we make the assumption that diffusion of the A cation is the rate-determining step in the SH. This assumption is not unreasonable, given the time scale mostly seen for SH (minutes to hours), while, if halide diffusion were rate-determining, SH would be expected to be faster.

Diffusion coefficients for halides in any one HaP vary over orders of magnitude in the literature.^31^ Many factors can affect halide diffusion in HaPs, such as halide vacancy concentration, illumination, the presence of water, and resulting proton diffusion, which might be confused with halide diffusion in some measurements.^40^ The most studied HaP is MAPI, and for this compound, the best order of magnitude estimate for iodide diffusion is 10^–12^–10^–13^ cm^2^ s^–1^ (for dry MAPI in the dark; cf. Table 2 in ref (31)). Computational theory gave for CsPI a value of ∼10^–13^ cm^2^ s^–1^.^41^ We have only been able to find indirect measurements for I^–^ diffusion in FAPI. Diffusion coefficients of 3 × 10^–13^ cm^2^ s^–1^ for FAPI, an order of magnitude smaller than that for MAPI, were deduced from impedance measurements.^11^ While they interpreted these values as probable diffusion coefficients of FA^+^ or MA^+^, it seems much more likely that they represent I^–^ diffusion. The reasons are that the values agree with those expected for halide diffusion and because they are much higher than those expected for A cation diffusion. A theoretical study predicted activation energies of diffusion of I^–^ vacancies in MAPI and FAPI, which showed comparable values for both HaPs (slightly higher for FAPI).^42^ As noted already, another theoretical study^39^ gave energy barriers for iodide migration decreasing as MA > FA > Cs, meaning that iodide diffusion is expected to be fastest in Cs and slowest in MA. The bottom line is that there is not much information in the literature that makes it possible to compare iodide diffusion between the three A cation perovskites, and what there is, is conflicting. No less important, in the absence of a microscopic mechanism for SH (e.g., is an ion displaced but remains close to its origin or does it move relatively far away), it is not clear if faster ion diffusion is beneficial or detrimental to SH, although it seems obvious that some ion diffusion is necessary.

The dipole moments of A-site cations in CsPI, FAPI, and MAPI are 0.00, 0.21, and 2.29 D, respectively;^43^ thus, there is an anticorrelation between these dipole moments and the SH kinetics. We have already referred to the theoretical study of ref (39) where the interaction of the A cation’s dipole moment with halide vacancies for MAPI, for FAPI, and for CsPI was considered and used to calculate both iodide vacancy formation energies (MA < FA ≪ Cs—the large difference between FA and Cs suggests no correlation with our results) and activation energies of iodide migration, as already noted. Naturally, as is the case with lattice distortion, the A cation dipole moment can have more than one effect on HaP stability (e.g., there might be dipole–dipole interactions between neighboring MA cations that will be negligible or absent for FA and Cs).

Photodecomposition was analyzed in a comparative mass spectrometry study of the three iodides, APbI_3_ (A = MA, FA, and Cs), also considered here.^44^ The results show significant differences in photodamage products between these HaPs. A similar conclusion came from our one-photon confocal study of single crystals of the corresponding bromides, with a slightly different product distribution for the FA HaP.^17^ (Note that thermal and photodecomposition need not yield the same products, as reaction pathways may well differ.).

Cs as such obviously does not decompose and, with encapsulation, also does not readily react with the ambient environment. Thus, few decomposition pathways apart from the well-known photolysis of PbI_2_ are left.

MA quite readily decomposes, forming mainly methylamine and HI.^44^ In the present experiments, MAPI shows incomplete recovery even after a week of continuous SH measurement, suggesting some irreversible product(s) formation.

The thermal decomposition of FAPI is known to be significantly slower than for MAPI,^45,46^ with (the unstable) formamidine, HI, and, ultimately, triazine as products. Also, the mass spectrometry results^44^ showed a significantly lower emission of organic species in FAPI than in MAPI photodecomposition, which fits with the lower volatility of FA^+^ and faster recovery of FAPI than MAPI; it also agrees with our earlier results on bromides.^17^

Our results (Figure 1) show that FAPI is photodamaged, under the conditions of our experiments, slightly more readily (at lower laser power) than MAPI. We need to remember, though, that our samples are encapsulated in contrast to the case for thermal decomposition studies (where FA-HaPs will be more stable than MA-HaPs). We find a qualitative anticorrelation between our measured photostability and the SH kinetics of the three different iodide perovskites. Still, based on all the data available, there is no apparent link between these two properties.

We find that different A cations affect photodamage and self-healing in the type of polycrystalline thin films of Pb iodide perovskites used in device studies, conditions that are approximated by encapsulating the films. CsPI and FAPI healed much more quickly than MAPI and recovered close to completely even after what seemed catastrophic damage from the photoluminescence intensity (90–95% PL intensity decrease). CsPI showed, especially from 30 min after the photodamage, faster SH than FAPI. Differences in lattice distortion (octahedral tilting) in the A cations’ dipole moments or the ability to form H-bonds can be correlated (inversely for the last two causes) with the observed SH kinetics. Thus, these factors could be involved in the variations in SH that we find. The anticorrelation between the speed of SH and either the dipole moment strength or H-bonding ability could be understood in terms of minimizing the depth of the energy landscape features, which will facilitate recovery from photodamage by recombination of damage products. Naturally, an additional factor can be differences in degradation pathways that are open to the different HaPs due to the different A cations in them.

Our results explain the success of the mixed cation halide perovskite compositions in terms of device stability. Compositions with mostly Cs and FA prevail, as any damage during their use in solar cells will heal rapidly, and even severe damage can heal overnight. More generally, the faster the SH kinetics, the better the material for autonomous sustainable electronics. The chances for complete recovery from damage increase with a decrease in the time needed for SH. Considering the slightly faster initial SH of FAPI than of CsPI after severe damage (Figure 3c) but subsequent faster SH rate of CsPI, such differences in SH with time may also be an essential parameter for devices. For example, for PV cells, faster initial SH may be important during daylight illumination, where it limits the steady-state density of defects. At night, the greater extent of SH over longer times is likely to be more critical.

## Supporting Information Available

Experimental section, evaluation of temperature after damage, factors affecting the kinetics of self-healing, X-ray diffraction patterns, AFM measurements of photodamage, photoluminescence spectra and decay curves, and additional self-healing kinetics (PDF)

## References

[ref1] Tian J.; Xue Q.; Yao Q.; Li N.; Brabec C. J.; Yip H.-L. Inorganic Halide Perovskite Solar Cells: Progress and Challenges. Adv. Energy Mater. 2020, 10 (23), 2000183 10.1002/aenm.202000183.

[ref2] Saikia D.; Betal A.; Bera J.; Sahu S. Progress and Challenges of Halide Perovskite-Based Solar Cell- a Brief Review. Materials Science in Semiconductor Processing 2022, 150, 106953 10.1016/j.mssp.2022.106953.

[ref3] Lu M.; Zhang Y.; Wang S.; Guo J.; Yu W. W.; Rogach A. L. Metal Halide Perovskite Light-Emitting Devices: Promising Technology for Next-Generation Displays. Adv. Funct. Mater. 2019, 29 (30), 1902008 10.1002/adfm.201902008.

[ref4] Liu X.-K.; Xu W.; Bai S.; Jin Y.; Wang J.; Friend R. H.; Gao F. Metal Halide Perovskites for Light-Emitting Diodes. Nat. Mater. 2021, 20 (1), 10–21. 10.1038/s41563-020-0784-7. 32929252

[ref5] Jeong D.-N.; Yang J.-M.; Park N.-G. Roadmap on Halide Perovskite and Related Devices. Nanotechnology 2020, 31 (15), 152001 10.1088/1361-6528/ab59ed. 31751955

[ref6] Liu F.; Wu R.; Wei J.; Nie W.; Mohite A. D.; Brovelli S.; Manna L.; Li H. Recent Progress in Halide Perovskite Radiation Detectors for Gamma-Ray Spectroscopy. ACS Energy Lett. 2022, 7 (3), 1066–1085. 10.1021/acsenergylett.2c00031.

[ref7] Liu F.; Wu R.; Zeng Y.; Wei J.; Li H.; Manna L.; Mohite A. D. Halide Perovskites and Perovskite Related Materials for Particle Radiation Detection. Nanoscale 2022, 14 (18), 6743–6760. 10.1039/D2NR01292H. 35470846

[ref8] Zheng Y.; Yang S. Stabilization Techniques of Lead Halide Perovskite for Photovoltaic Applications. Solar RRL 2022, 6 (1), 2100710 10.1002/solr.202100710.

[ref9] Zhao X.; Park N.-G. Stability Issues on Perovskite Solar Cells. Photonics 2015, 2 (4), 1139–1151. 10.3390/photonics2041139.

[ref10] Boyd C. C.; Cheacharoen R.; Leijtens T.; McGehee M. D. Understanding Degradation Mechanisms and Improving Stability of Perovskite Photovoltaics. Chem. Rev. 2019, 119 (5), 3418–3451. 10.1021/acs.chemrev.8b00336. 30444609

[ref11] Bag M.; Renna L. A.; Adhikari R. Y.; Karak S.; Liu F.; Lahti P. M.; Russell T. P.; Tuominen M. T.; Venkataraman D. Kinetics of Ion Transport in Perovskite Active Layers and Its Implications for Active Layer Stability. J. Am. Chem. Soc. 2015, 137 (40), 13130–13137. 10.1021/jacs.5b08535. 26414066

[ref12] Nie W.; Blancon J.-C.; Neukirch A. J.; Appavoo K.; Tsai H.; Chhowalla M.; Alam M. A.; Sfeir M. Y.; Katan C.; Even J.; Tretiak S.; Crochet J. J.; Gupta G.; Mohite A. D. Light-Activated Photocurrent Degradation and Self-Healing in Perovskite Solar Cells. Nat. Commun. 2016, 7 (1), 11574 10.1038/ncomms11574. 27181192 PMC4873646

[ref13] Lang F.; Nickel N. H.; Bundesmann J.; Seidel S.; Denker A.; Albrecht S.; Brus V. V.; Rappich J.; Rech B.; Landi G.; Neitzert H. C. Radiation Hardness and Self-Healing of Perovskite Solar Cells. Adv. Mater. 2016, 28 (39), 8726–8731. 10.1002/adma.201603326. 27529814

[ref14] Finkenauer B. P.; Akriti; Ma K.; Dou L. Degradation and Self-Healing in Perovskite Solar Cells. ACS Appl. Mater. Interfaces 2022, 14 (21), 24073–24088. 10.1021/acsami.2c01925. 35588005

[ref15] Khenkin M. V.; K M A.; Visoly-Fisher I.; Galagan Y.; Di Giacomo F.; Patil B. R.; Sherafatipour G.; Turkovic V.; Rubahn H.-G.; Madsen M.; Merckx T.; Uytterhoeven G.; Bastos J. P. A.; Aernouts T.; Brunetti F.; Lira-Cantu M.; Katz E. A. Reconsidering Figures of Merit for Performance and Stability of Perovskite Photovoltaics. Energy Environ. Sci. 2018, 11 (4), 739–743. 10.1039/C7EE02956J.

[ref16] Ceratti D. R.; Rakita Y.; Cremonesi L.; Tenne R.; Kalchenko V.; Elbaum M.; Oron D.; Potenza M. A. C.; Hodes G.; Cahen D. Self-Healing Inside APbBr _3_ Halide Perovskite Crystals. Adv. Mater. 2018, 30 (10), 1706273 10.1002/adma.201706273. 29328524

[ref17] Ceratti D. R.; Cohen A. V.; Tenne R.; Rakita Y.; Snarski L.; Jasti N. P.; Cremonesi L.; Cohen R.; Weitman M.; Rosenhek-Goldian I.; Kaplan-Ashiri I.; Bendikov T.; Kalchenko V.; Elbaum M.; Potenza M. A. C.; Kronik L.; Hodes G.; Cahen D. The Pursuit of Stability in Halide Perovskites: The Monovalent Cation and the Key for Surface and Bulk Self-Healing. Mater. Horiz. 2021, 8 (5), 1570–1586. 10.1039/D1MH00006C. 34846465

[ref18] Aharon S.; Ceratti D. R.; Jasti N. P.; Cremonesi L.; Feldman Y.; Potenza M. A. C.; Hodes G.; Cahen D. 2D Pb-Halide Perovskites Can Self-Heal Photodamage Better than 3D Ones. Adv. Funct. Mater. 2022, 32 (24), 2113354 10.1002/adfm.202113354.

[ref19] Ceratti D. R.; Tenne R.; Bartezzaghi A.; Cremonesi L.; Segev L.; Kalchenko V.; Oron D.; Potenza M. A. C.; Hodes G.; Cahen D. Self-Healing and Light-Soaking in MAPbI3: The Effect of H2O. Adv. Mater. 2022, 34 (35), 2110239 10.1002/adma.202110239. 35731235

[ref20] Yadavalli S. K.; Dai Z.; Zhou H.; Zhou Y.; Padture N. P. Facile crack-healing in organic-inorganic halide perovskite thin films. Acta Mater. 2020, 187, 112–121. 10.1016/j.actamat.2020.01.040.

[ref21] Al-Handawi M. B.; Dushaq G.; Commins P.; Karothu D. P.; Rasras M.; Catalano L.; Naumov P. Autonomous Reconstitution of Fractured Hybrid Perovskite Single Crystals. Adv. Mater. 2022, 34 (19), 2109374 10.1002/adma.202109374. 35234306

[ref22] Ma F.; Li J.; Li W.; Lin N.; Wang L.; Qiao J. Stable α/δ Phase Junction of Formamidinium Lead Iodide Perovskites for Enhanced near-Infrared Emission. Chem. Sci. 2017, 8 (1), 800–805. 10.1039/C6SC03542F. 28451230 PMC5301192

[ref23] Binek A.; Hanusch F. C.; Docampo P.; Bein T. Stabilization of the Trigonal High-Temperature Phase of Formamidinium Lead Iodide. J. Phys. Chem. Lett. 2015, 6 (7), 1249–1253. 10.1021/acs.jpclett.5b00380. 26262982

[ref24] Zhao B.; Jin S.-F.; Huang S.; Liu N.; Ma J.-Y.; Xue D.-J.; Han Q.; Ding J.; Ge Q.-Q.; Feng Y.; Hu J.-S. Thermodynamically Stable Orthorhombic γ-CsPbI3 Thin Films for High-Performance Photovoltaics. J. Am. Chem. Soc. 2018, 140 (37), 11716–11725. 10.1021/jacs.8b06050. 30153411

[ref25] Shi L.; Young T. L.; Kim J.; Sheng Y.; Wang L.; Chen Y.; Feng Z.; Keevers M. J.; Hao X.; Verlinden P. J.; Green M. A.; Ho-Baillie A. W. Y. Accelerated Lifetime Testing of Organic–Inorganic Perovskite Solar Cells Encapsulated by Polyisobutylene. ACS Appl. Mater. Interfaces 2017, 9 (30), 25073–25081. 10.1021/acsami.7b07625. 28700216

[ref26] Gaulding E. A.; Louks A. E.; Yang M.; Tirawat R.; Wilson M. J.; Shaw L. K.; Silverman T. J.; Luther J. M.; Palmstrom A. F.; Berry J. J.; Reese M. O. Package Development for Reliability Testing of Perovskites. ACS Energy Lett. 2022, 7 (8), 2641–2645. 10.1021/acsenergylett.2c01168.

[ref27] Masi S.; Gualdrón-Reyes A. F.; Mora-Seró I. Stabilization of Black Perovskite Phase in FAPbI_3_ and CsPbI_3_. ACS Energy Lett. 2020, 5 (6), 1974–1985. 10.1021/acsenergylett.0c00801.

[ref28] Abney M. K.; Suri M.; Shah T.; Deepak F. L.; Korgel B. A. Reversible Light-Induced Enhancement of Photoluminescence Lifetime and Intensity in Perovskite-Phase CsPbI _3_ Nanocrystals. J. Phys. Chem. C 2022, 126 (30), 12712–12720. 10.1021/acs.jpcc.2c04305.

[ref29] Duan L.; Zhang H.; Liu M.; Grätzel M.; Luo J. Phase-Pure γ-CsPbI3 for Efficient Inorganic Perovskite Solar Cells. ACS Energy Lett. 2022, 7 (9), 2911–2918. 10.1021/acsenergylett.2c01219.

[ref30] Zhang H.; Chen Z.; Qin M.; Ren Z.; Liu K.; Huang J.; Shen D.; Wu Z.; Zhang Y.; Hao J.; Lee C.; Lu X.; Zheng Z.; Yu W.; Li G. Multifunctional Crosslinking-Enabled Strain-Regulating Crystallization for Stable, Efficient α-FAPbI3-Based Perovskite Solar Cells. Adv. Mater. 2021, 33 (29), 2008487 10.1002/adma.202008487. 34085738

[ref31] Saidaminov M. I.; Kim J.; Jain A.; Quintero-Bermudez R.; Tan H.; Long G.; Tan F.; Johnston A.; Zhao Y.; Voznyy O.; Sargent E. H. Suppression of Atomic Vacancies via Incorporation of Isovalent Small Ions to Increase the Stability of Halide Perovskite Solar Cells in Ambient Air. Nat. Energy 2018, 3 (8), 648–654. 10.1038/s41560-018-0192-2.

[ref32] Rakita Y.; Lubomirsky I.; Cahen D. When Defects Become ‘Dynamic’: Halide Perovskites: A New Window on Materials?. Mater. Horiz. 2019, 6 (7), 1297–1305. 10.1039/C9MH00606K.

[ref33] Kumar S.; Hodes G.; Cahen D. Defects in Halide Perovskites: The Lattice as a Boojum?. MRS Bull. 2020, 45 (6), 478–484. 10.1557/mrs.2020.146.

[ref34] Cohen A.; Brenner T. M.; Klarbring J.; Sharma R.; Fabini D. H.; Korobko R.; Nayak P. K.; Hellman O.; Yaffe O. Diverging Expressions of Anharmonicity in Halide Perovskites. Adv. Mater. 2022, 34 (14), 2107932 10.1002/adma.202107932. 35076969

[ref35] Han G.; Hadi H. D.; Bruno A.; Kulkarni S. A.; Koh T. M.; Wong L. H.; Soci C.; Mathews N.; Zhang S.; Mhaisalkar S. G. Additive Selection Strategy for High Performance Perovskite Photovoltaics. J. Phys. Chem. C 2018, 122 (25), 13884–13893. 10.1021/acs.jpcc.8b00980.

[ref36] Zheng X.; Wu C.; Jha S. K.; Li Z.; Zhu K.; Priya S. Improved Phase Stability of Formamidinium Lead Triiodide Perovskite by Strain Relaxation. ACS Energy Lett. 2016, 1 (5), 1014–1020. 10.1021/acsenergylett.6b00457.

[ref37] Svane K. L.; Forse A. C.; Grey C. P.; Kieslich G.; Cheetham A. K.; Walsh A.; Butler K. T. How Strong Is the Hydrogen Bond in Hybrid Perovskites?. J. Phys. Chem. Lett. 2017, 8 (24), 6154–6159. 10.1021/acs.jpclett.7b03106. 29216715 PMC5765532

[ref38] Senocrate A.; Moudrakovski I.; Acartürk T.; Merkle R.; Kim G. Y.; Starke U.; Grätzel M.; Maier J. Slow CH _3_ NH _3_^+^ Diffusion in CH_3_NH_3_PbI_3_ under Light Measured by Solid-State NMR and Tracer Diffusion. J. Phys. Chem. C 2018, 122 (38), 21803–21806. 10.1021/acs.jpcc.8b06814.

[ref39] Pazoki M.; Wolf M. J.; Edvinsson T.; Kullgren J. Vacancy dipole interactions and the correlation with monovalent cation dependent ion movement in lead halide perovskite solar cell materials. Nano Energy 2017, 38, 537–543. 10.1016/j.nanoen.2017.06.024.

[ref40] Ceratti D. R.; Zohar A.; Kozlov R.; Dong H.; Uraltsev G.; Girshevitz O.; Pinkas I.; Avram L.; Hodes G.; Cahen D. Eppur Si Muove: Proton Diffusion in Halide Perovskite Single Crystals. Adv. Mater. 2020, 32 (46), 2002467 10.1002/adma.202002467. 33048452

[ref41] Balestra S. R. G.; Vicent-Luna J. M.; Calero S.; Tao S.; Anta J. A. Efficient Modelling of Ion Structure and Dynamics in Inorganic Metal Halide Perovskites. J. Mater. Chem. A 2020, 8 (23), 11824–11836. 10.1039/D0TA03200J.

[ref42] Haruyama J.; Sodeyama K.; Han L.; Tateyama Y. First-Principles Study of Ion Diffusion in Perovskite Solar Cell Sensitizers. J. Am. Chem. Soc. 2015, 137 (32), 10048–10051. 10.1021/jacs.5b03615. 26258577

[ref43] Gets D. S.; Verkhogliadov G. A.; Danilovskiy E. Y.; Baranov A. I.; Makarov S. V.; Zakhidov A. A. Dipolar Cation Accumulation at the Interfaces of Perovskite Light-Emitting Solar Cells. J. Mater. Chem. C 2020, 8 (47), 16992–16999. 10.1039/D0TC02654A.

[ref44] Song Z.; Wang C.; Phillips A. B.; Grice C. R.; Zhao D.; Yu Y.; Chen C.; Li C.; Yin X.; Ellingson R. J.; Heben M. J.; Yan Y. Probing the Origins of Photodegradation in Organic–Inorganic Metal Halide Perovskites with Time-Resolved Mass Spectrometry. Sustainable Energy Fuels 2018, 2 (11), 2460–2467. 10.1039/C8SE00358K.

[ref45] Juarez-Perez E. J.; Ono L. K.; Qi Y. Thermal Degradation of Formamidinium Based Lead Halide Perovskites into *Sym* -Triazine and Hydrogen Cyanide Observed by Coupled Thermogravimetry-Mass Spectrometry Analysis. J. Mater. Chem. A 2019, 7 (28), 16912–16919. 10.1039/C9TA06058H.

[ref46] Luongo A.; Brunetti B.; Vecchio Ciprioti S.; Ciccioli A.; Latini A. Thermodynamic and Kinetic Aspects of Formamidinium Lead Iodide Thermal Decomposition. J. Phys. Chem. C 2021, 125 (40), 21851–21861. 10.1021/acs.jpcc.1c06729. PMC8521522 34676017

