# Supplementary material for: A-Site Cation Dependence of Self-Healing in Polycrystalline APbI3 Perovskite Films
Source: ACS Energy Lett. 2023 May 3;8(5):2447–55. doi: 10.1021/acsenergylett.3c00017 (PMC10189583; doi:10.1021/acsenergylett.3c00017)
Supplement: Supplementary file 1 — nz3c00017_si_001.pdf [file nz3c00017_si_001.pdf]

Supplementary information

for

A-site Cation Dependence of Self-Healing in Polycrystalline *A*PbI_3 Perovskite Films

Pallavi Singh[†1], Yahel Soffer[†2], Davide Raffaele Ceratti[3], Michael Elbaum,[4] Dan Oron[1*], Gary Hodes[1*], David Cahen[1*]

[1]Dept. of Molecular Chemistry and Materials Science, Weizmann Institute of Science, 7610001, Israel
[2] Dept. of Physics of Complex Systems, Weizmann Institute of Science, Rehovot 76100, Israel;
[3]Institut Photovoltaïque d'Ile-de-France (IPVF) – 91120 Palaiseau, France
[4] Dept. of Chemical & Biological Physics, Weizmann Institute of Science, 7610001, Israel
[†]Contributed equally to this work. [*]Corresponding authors

This supplementary information file contains sections discussing thin film fabrication, their encapsulation, measurement parameters used in XRD, photo-damage and self-healing (confocal/wide field) experiments, in this study. Also, we present experimental data, which are discussed in the main text.

Experimental section:

SI 1. Thin film fabrication and encapsulation
SI 2. X-Ray diffraction measurements of the perovskite thin films
SI 3. One-Photon Confocal Microscopy: Imaging and photo-bleaching conditions
SI 4. Evaluation of temperature after damage
SI 5. Energy deposited in the films
SI 6. Time-resolved PL on encapsulated CsPI films
SI 7 AFM measurements on MAPI films
SI 8. Factors affecting kinetics of self-healing reaction

Supplementary Schemes, Figures and Tables:

Scheme S1 Schematic of sample architecture of encapsulated MAPI used for AFM measurements
Figure S1 Unencapsulated FAPI and CsPI films in ambient showing transformation to yellow photo inactive phase at room temperature
Figure S2 Programmed illumination pattern used for the FRAP studies with 1P- confocal microscopy.
Figure S3 Diffraction pattern of room temperature photoactive $\beta-$ $MAPbI_3$ (MAPI) phase.
Figure S4 Diffraction pattern of high temperature photoactive α − $FAPbI_3$ (FAPI) phase
Figure S5 Diffraction pattern of low temperature photoactive $\gamma-$ $CsPbI_3$ (CsPI) phase (major fraction) along with low temperature photo-inactive orthorhombic δ−$CsPbI_3$ phase.
Figure S6 Uniformity in photo-damage threshold and photo-response (photo-damage extent) in $MAPbI_3$, HT α − $FAPbI_3$ and LT $\gamma-$ $CsPbI_3$ thin films.
Figure S7 AFM measurement of photo-damaged surface of MAPI film.
Figure S8 Representative photoluminescence spectra and time-resolved PL decay curves of damaged and undamaged areas of a CsPI film.
Figure S9 Thin films of α-$FAPbI_3$ showing photo-brightening in the periphery of ROIs.

Figure S10 SH kinetics in additional encapsulated polycrystalline MAPI, FAPI and CsPI films using 488 nm CW laser.
Figure S11 SH kinetics in additional encapsulated polycrystalline MAPI, FAPI and CsPI films using 405 nm CW laser.
Figure S12 SH kinetics in additional encapsulated polycrystalline MAPI samples using 405 nm CW laser.

Figure S13 PL spectra showing increase in PL intensity and no change in spectral pattern for the damaged ROI in $MAPbI_3$ thin film. PL spectrum of reference and healed ROI.
Figure S14 PL spectra show increase in PL intensity and no change in spectral pattern for the damaged ROI in high temperature photoactive α-$FAPbI_3$ thin film.
Figure S15 PL spectra show increase in PL intensity of photo-brightened area compared to reference area with no change in spectral pattern in low temperature photoactive γ-$CsPbI_3$ thin film.
Figure S16 SH kinetics of unencapsulated, single crystals of $CsPbBr_3$, $FAPbBr_3$ and $MAPbBr_3$ at the near surface of the crystals (using 1-photon excitation laser).
Figure S17 As Figure SI 16, but using 2-photon laser to damage inside the macroscopic crystals far from the surface.

Table S1 The LP% corresponding laser powers and power densities for imaging/photo-damage studies in thin films using confocal laser scanning microscopy.

Experimental section:

SI 1. Thin film fabrication and encapsulation

1.1. Chemicals:

Lead iodide and cesium iodide were purchased from Sigma Aldrich. Organic cation salts such as methylammonium iodide and formamidinium iodide were purchased from Greatcell Solar. Solvents like anhydrous dimethyl formamide (DMF) and anhydrous dimethyl sulfoxide (DMSO) were purchased from Sigma Aldrich. The polymer polyisobutylene was purchased from J&K scientific. All the materials were used as received. Microscope coverslip (number 1.5H) with thickness 175±5 µm was used a substrate and purchased from Marienfeld.

1.2. Synthesis of Perovskite thin films:

We use 175 ± 5 µm thick coverslips as a substrate to avoid aberrations as our 60x oil (NA 1.4) immersion confocal microscope objective was corrected for this thickness (this made the SH study of encapsulated sample possible). Substrates were cleaned sequentially with acetone, ethanol and de-ionized water for 10 minutes each followed by drying with a nitrogen gun. They were then treated with UV-ozone to destroy organic contaminants on the surface and to increase hydrophilicity for improved surface coverage. The substrates were immediately transferred to a glove box.

All subsequent processes, including solution preparations, thin film deposition, annealing, as well as encapsulation, were done inside a N_2-filled glove box maintained at less than 0.1% H_2O and 0.1 ppm O_2. Prior to deposition, all solutions were stirred at 70 ºC for 2-3 hours and then cooled to RT.

$MAPbI_3$ thin film: 1.4 M solution was prepared using 1:1 molar ratio of MAI and PbI_2 in DMF:DMSO (9:1). The deposition parameter was 4000 rpm with acceleration 4000 rpm/sec for 30 sec. 150 µL chlorobenzene antisolvent was introduced after 10 sec from the start of spinning. The films were annealed at 65 ºC for 1 min followed by 100 °C for 10 min.

α-$FAPbI_3$ thin film: Deposition parameters and solution concentration of FAI and PbI_2 are the same as for $MAPbI_3$: however, just before using the solution for deposition, HI was added. The films were annealed at 70 ºC for 1min followed by 170 ºC for 25 min.

There is no indication of the formation of the low temperature photo inactive δ-$FAPbI_3$ phase as the near-infrared PL characteristic of the α– phase fully recovers. We note that photoactive FAPI phase formation at RT was already reported with the help of a formamidinium-SCN additive that led to the formation of an FAI-SCN-PbI_2 intermediate, from which the SCN was expelled after antisolvent injection.[1]

γ-$CsPbI_3$ thin film: We followed the same procedure reported by Zhao et al.[2]. 500 µL stock solution was prepared and just before spin coating 16 µL of HI containing stabilizer was added in the stock solution followed by addition of 0.1µL of deionized water (0.02V% of DMF). Films were coated at 1000 rpm for 10 sec, which further increased to 5000 rpm for 25 sec. This was followed by chlorobenzene antisolvent dispensed 10 sec before the end of the spin coating. Samples immediately turned black with a glossy finish when placed on a hot plate at 100 ºC for 5 min. The CsPI may contain some dimethylammonium from hydrolysis of DMF used in the preparation.[3]

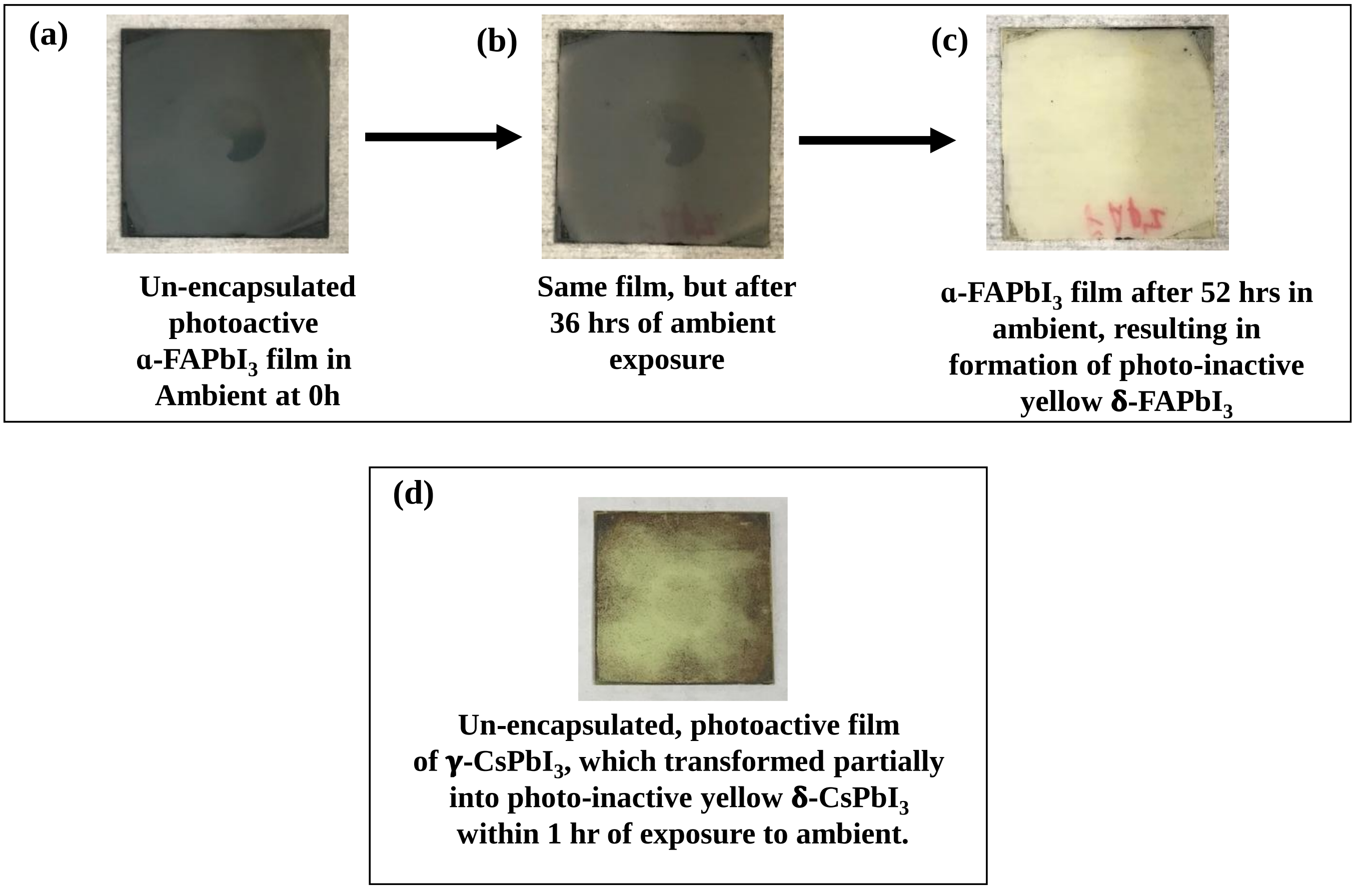

Figure S1. Unencapsulated FAPI and CsPI films in ambient showing transformation of black photoactive phase to yellow (shown as bright) photo inactive phase at room temperature; (a-c) showing changes in FAPI where transformation took nearly 2-3 days, (d) showing changes in CsPI where transformation takes place in less than an hour.

1.3. Sample Encapsulation:

All steps of sample encapsulation were performed inside a dry N_2-filled glove box. After deposition and annealing of the perovskite, the edges of the substrate were cleaned from all directions with a blade as seen scheme S1a (step 3). Nitrogen gas was passed over the substrate to remove any particles from the surface. We avoid the use of polar polymers as they have a tendency to react with the polar photo-damaged products as an effect of photo-damage created by higher laser power densities; for example, corresponding amines of organic quaternary ammonium salts react with the carbonyl group of PMMA and PVA. Therefore, we have selected a non-polar PIB polymer as an encapsulant and prepared its solution in a suitable non-polar solvent toluene with concentration 12mg/mL. After complete dissolution of polymer in the solvent (5-6 hrs), the polymer solution was drop-casted onto the perovskite film and annealed at 100 °C for 2-3 min, then cooled and the process was repeated for a more reliably blocking film (Scheme 1a, step 4). To further block the effects of H_2O and O_2, 5 min epoxy as an edge sealant was applied on the cleaned edges followed by placing the coverslip on the top (Scheme 1a, step 5 and 6). The sample was pressed gently to uniformly distribute sealant and also to avoid any bubbles in the edge sealing layer. Further, the whole sample was clipped with a binder (paper) clip to ensure tight packing between the two coverslips. The curing time for

the 5 min epoxy we used is 1 hr, whereas it takes 16 hours to reach full bond strength and after that, the sample is ready for self-healing measurements (Scheme 1b). Note the encapsulated sample is exposed to the laser irradiation on the side opposite the polymer side); this further reduces any interaction of photo-damaged product with any of the sample components such as PIB polymer and 5 min epoxy edge sealant. Both polymer and edge sealant act as a double protection barrier to avoid any influence of ambient on the polycrystalline film during the course of measurement.

SI 2. X-Ray Diffraction measurement of the perovskite thin film:

The diffraction patterns were recorded using a Rigaku TTRAX X-ray diffractometer at room temperature using Cu-Kα radiation. The data was acquired in the range 5–60° with a scan rate of 1°/min and a step size of 0.01°. Structural analysis by XRD served to check for phase purity and find the presence of secondary phases.
Sample preparation for XRD: Partially encapsulated samples with a layer of PIB polymer on the perovskite films were used for XRD measurement. The polymer was spin-coated at 2000 rpm for 30 sec and annealed at 100 °C for 2-3 minutes. The PIB layer protected against the ambient sufficiently well to carry out the XRD measurements.

SI 3. One-photon confocal microscopy: Imaging and photo-bleaching conditions:

1. FRAP studies were carried out on an Olympus Fluoview confocal microscope (BX50WI) equipped with several lasers as excitation sources. A supra-bandgap laser beam (488 nm) illuminated the sample surface and the resulting photoluminescence (PL) signal was measured. Care was taken to work at sufficiently low beam intensity so as not to cause any measurable damage. The result defines the "non-damage" baseline PL. The laser wavelength was selected as the absorption coefficient at this wavelength is sufficiently high so that most of the absorption occurs in the top 100 nm of the layer.
   For effective gathering of the diffused PL from the surface of the polycrystalline films, we used a 60x oil immersion objective with 1.4 numerical aperture. The area of exposed ROIs is the same in all cases, irrespective of power density and material composition.
   The laser was raster-scanned over the sample with a scan rate of 2.71 sec/scan over 512×512 pixels with a dwell time per pixel of 7.2 µs, which is constant for all compositions. For all perovskite compositions we use the same power density of 0.04×10^5 W/cm^2 for imaging, which is only 1% of total laser power of 488 nm. We used a 700 nm barrier filter to block any 488 nm reflected light. The confocal aperture positioned in front of the detector, which defines the analysis volume of the sample, was 300 μm in diameter.
2. The sample surface was exposed to 1-2 orders of magnitude higher laser powers than the imaging power to cause localized photo-damage in the regions of interest (ROIs appear as circular spots in Figures 2 & 3) with the help of a programmed mask (Figure SI1). This mask allows the setting of laser power percentages (18%-38% including 1%), and we calculated corresponding power densities, which vary from 0.04×10^5 W/cm^2 to 1.55×10^5 W/cm^2. We found 1.55×10^5 W/cm^2 suffices to cause 90-100% PL loss in all cases; therefore, this power density was set as a maximum in the mask. The beam diameter was 0.25 µm, its area size 0.005 µm^2. The study involving damage threshold comparison was limited to this step only; however, the study of healing kinetics involved the following additional step.
3. The study of recovery kinetics, which starts a few seconds after the pulse that causes the photo-damage, involved tracking the PL from the damaged ROIs over time using the low laser power density used originally for imaging. The ambient temperature was maintained in the range 24-25º C for reasons of instrumental stability.

4. All the above steps (before and after and photo-damage) were followed by acquisition of the PL spectrum (before and immediately after, as well as during recovery of the photo-damage) to check if the material had changed (decomposition or phase transformation). Because of instrument limitation, we measured the PL spectrum of MAPI and CsPI with the same setup as that used for the PL intensity recovery, but the PL spectrum of FAPI was measured ex-situ.

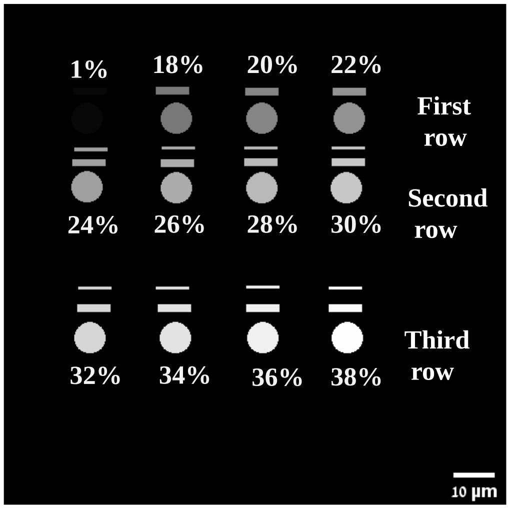

Figure S2: Programmed illumination pattern used for the FRAP studies with 1P- confocal microscopy.

***Table S1*:** The LP% corresponding laser powers and power densities.

Laser power %	Corresponding laser power (μW)	beam area (μm^2)	Power density (10^5 W/cm^2)
1	10	0.25	0.04
18	178		0.71
20	194		0.77
22	216		0.86
24	239		0.95
26	258		1.03
28	277		1.11
30	300		1.20
32	324		1.30
34	344		1.38
36	365		1.46
38	388		1.55

SI 4. Evaluation of temperature after damage

We calculate the temperature change in the film at the laser focus at the end of the bleaching cycle. As described in the main text the laser light is absorbed completely by the perovskite film. In the following calculation, we consider the thermal parameters of the glass only; glass conducts heat better than PIB ($\sim 0.4\frac{W}{mk}$) and FA/MA/$CsPbI_3$ ($\sim 0.5\frac{W}{mK}$)[4,5], and since glass is 2 orders of magnitude thicker in our specimen, heat will diffuse mostly through the glass. The temperature change follows the diffusion equation:

$$\nabla^2 T + \frac{Q}{\rho C_p} = \frac{1}{\alpha}\frac{\partial T}{\partial t}$$

$$\alpha = \frac{k}{\rho C_p}$$

where T is the temperature, ΔT is the difference in temperature between the local temperature and the background (pre-bleaching temperature), α is the thermal diffusivity, t is time, ρ is the density, C_p is the heat capacity, and k is the thermal conductivity. For glass the value of C_p, ρ and k is given below:[6]

$$C_p = 840\frac{J}{g\cdot k}; \rho = 2.5\frac{g}{cm^{-3}}; k = 1.05\ \frac{W}{m\cdot k}$$

$$=> \alpha = \frac{k}{\rho C_p} = \frac{1.05\cdot 10^{-2}\frac{W}{cm\cdot k}}{840\frac{J}{g\cdot k}\cdot 2.5\frac{g}{cm^{-3}}} = \ 5\cdot 10^{-6}\frac{cm^2}{s}$$

The solution of the heat equation for heat, released over time, and with some spatial distribution, is:

$$\Delta T(x,y,z,t) = \int dz' \int dy' \int dx' \int_0^t dt' \frac{Q(x',y',z',t')}{\rho C_p} \cdot \frac{1}{\left(4\pi\alpha(t-t')\right)^{\frac{3}{2}}} \exp\left(-\frac{((x-x')^2+(y-y')^2+(z-z')^2)}{4\alpha(t-t')}\right)$$

where $Q(x',y',z',t')$, the heat source, can be described as a gaussian distribution with P the energy absorbed by the film per unit time.

$$Q(x',y',z') = \frac{P}{(2\pi)^{\frac{3}{2}}}\frac{1}{\sigma_{x'}\sigma_{y'}\sigma_{z'}}\ \exp\left(-\left(\frac{x'^2}{2\sigma_{x'}^2}+\frac{y'^2}{2\sigma_{y'}^2}+\frac{z'^2}{2\sigma_{z'}^2}\right)\right))$$

To simplify the integral, we can describe the heat as if it originated from a point source, and after a period of time τ, it diffused to give this Gaussian profile of heat. Following the derivation from the SI of Ceratti et al.[7], diffusion to a Gaussian profile having $\sigma = 2.5\ \mu m$, will take 6.8 ms.

$$\tau = \frac{\sigma^2}{2\alpha} = \frac{(2.5)^2}{2\cdot 4.6\cdot 10^2} s = 6.8\ ms$$

Now the distribution of energy, instead of having a gaussian profile that has diffused time t', will have a point distribution of time $t' - \tau$.

We assume the heat was injected at a point ($x = y = z = 0$) and that the power absorbed by the film is constant during the bleaching time.

$$\Delta T(x, y, z, t) = \int_{-\tau}^{-\tau+t_{bleaching}} dt' \frac{P(t')}{\rho C_p} * \frac{1}{\left(4\pi\alpha(t-t')\right)^{\frac{3}{2}}} \exp\left(-\frac{((x-x')^2 + (y-y')^2 + (z-z')^2)}{4\alpha(t-t')}\right)$$

$$\Delta T(0,0,0, t) = \int_{-\tau}^{-\tau+t_{bleaching}} dt' \frac{P}{\rho C_p} \frac{1}{\left(4\pi\alpha(t-t')\right)^{\frac{3}{2}}}$$

Assuming that the maximum temperature change is reached at the end of the bleaching cycle, so $t = t_{bleaching}$, we get:

$$\Delta T\left(0,0,0, t_{bleaching}\right) = 2\left(\frac{1}{\left(t_{bleaching} - (-\tau + t_{bleaching})\right)^{\frac{1}{2}}} - \frac{1}{\left(t_{bleaching} - (-\tau)\right)^{\frac{1}{2}}}\right) \frac{P}{\rho C_p (4\pi\alpha)^{\frac{3}{2}}}$$

$P = 1\ mW$, $t_{bleaching} = 7\ \mu s$

$$\Delta T\left(0,0,0, t_{bleaching}\right) = 2\left(\frac{1}{(6.8 \cdot 10^{-3} s)^{\frac{1}{2}}} - \frac{1}{(6.8 \cdot 10^{-3} + 7 \cdot 10^{-6} s)^{\frac{1}{2}}}\right) \cdot \frac{1 \cdot 10^{-3} W}{2268 \frac{J}{cm^{-3} \cdot k}} \cdot \frac{1}{\left(4\pi \cdot 4.6 \cdot 10^{-6} \frac{cm^2}{s}\right)^{\frac{3}{2}}}$$

$$\Delta T = 0.3k$$

Thus, the maximal change in temperature at the end of the bleaching cycle (with 488 nm laser light) is less than 1 degree; therefore, under these experimental conditions, we inspect direct optical damage, and thermal effects can be neglected.

SI 5. Energy deposited in the films

The energy deposited per unit area was calculated as the peak power of the gaussian beam, I_{peak} , multiplied by the duration of the bleaching cycle, Δt. To obtain the experimental point spread function (PSF) of the system, we measured fluorescent spheres. We used the same scanning conditions as those used for SH measurements, to record fluorescent beads (F8811, Thermofisher) 180 nm radius (σ), with oversampling of 100 nm steps. Assuming a 2D gaussian beam, we got a beam σ of 280 nm.

$$Power = \frac{1}{2\pi\sigma^2} \exp\left(-\frac{((x)^2 + (y)^2)}{2\sigma^2}\right) => I_{peak} = \frac{Power}{2\pi\sigma^2}$$

where $Power$ is the power we measured of the whole beam area, $\pi\sigma^2$ (values of measured power are presented in Table S 1). At 1 mW, 100% laser power, the energy per unit area deposited on the film at the end of a bleaching cycle is:

$$Energy\ areal\ density = \frac{10^{-3}W}{((3\cdot 10^{-5})^2 2\pi)cm^{-2}} \cdot 10^{-5}\,\text{sec} = 2\ J/cm^2$$

SI 6. Time-resolved PL on CsPI films

Time-resolved photoluminescence (TRPL) of CsPI films was acquired using a 488 nm pulsed laser with 80 MHz repetition rate, with a single photon avalanche diode (SPAD) and time-correlated single photon counting, TCSPC, system.

Sample preparation for Time-resolved PL measurement: We use the same architecture that we use for SH studies. The encapsulated samples were exposed to a series of laser power densities using the 488 nm laser followed by measuring the lifetime.

SI 7. AFM measurements on MAPI films

The instrument, used for the depth profiling of photo-damaged circular ROIs, is an NT-MDT Prima, using a Universal head and 100 µm^2 scanner, in semi-contact mode, with an AC240 Olympus probe.

Sample preparation for AFM measurement: Samples were damaged using a 405 nm laser at maximum power, which leads to a higher temperature rise than with the 488 nm laser, as the latter was insufficient to cause AFM-measurable damage. A different sample architecture was used for AFM studies than for SH as that sample architecture was not suitable for AFM analysis. The sample was encapsulated inside the glove box and only a glass coverslip was used to cover the sample while edges were sealed with 5 minute Devcon epoxy glue. No (PIB) polymer cover was used as we were interest in observing the change in surface morphology after photo-damage. After a photo-damage event, encapsulation was broken and the sample was taken immediately for AFM analysis.

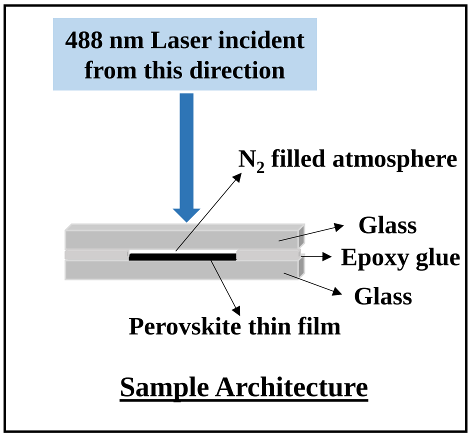

Scheme S1. Schematic of sample architecture of encapsulated MAPI used for AFM measurement; note that in this case no polyisobutylene polymer is used.

Section SI 8: Factors Affecting Kinetics of Self-healing Reaction

The kinetic reactions of species involved in an equilibrium of two (hypothetical) opposing reaction involving three compounds namely A, B and C (A can in our case be $APbI_3$ while B and C defects that are created by the photodamage and that do not require to be specifically identified for this discussion)

$$\mathrm{A + h\nu \leftrightarrow B + C}$$

Can be written as

$$\frac{d[A]}{dt} = -k_1(h\nu)[A] + k_2\,[B]\,[C]$$

$$\frac{d[B]}{dt} = \frac{d[C]}{dt} = -k_1(h\nu)[A] + k_2\,[B]\,[C]$$

where $[X]$ corresponds to the concentration of the specie X, $k_1(h\nu)$ a kinetic constant (that depends on the light intensity) and k_2 the kinetic of self-healing

Since at equilibrium

$$\frac{d[A]_{eq}}{dt} = \frac{d[B]_{eq}}{dt} = \frac{d[C]_{eq}}{dt} = 0$$

We can subtract

$$\frac{d[A]}{dt} - \frac{d[A]_{eq}}{dt} = -k_1(h\nu)\big([A] - [A]_{eq}\big) + k_2\,\big([B] - [B]_{eq}\big)\big([C] - [C]_{eq}\big)$$

$$\frac{d[B]}{dt} - \frac{d[B]_{eq}}{dt} = \frac{d[C]}{dt} - \frac{d[C]_{eq}}{dt} = +k_1(h\nu)\big([A] - [A]_{eq}\big) - k_2\,\big([B] - [B]_{eq}\big)\big([C] - [C]_{eq}\big)$$

That is

$$\frac{d[A]}{dt} = -k_1(h\nu)\Delta[A] + k_2\,\Delta[B]\,\Delta[C]$$

$$\frac{d[B]}{dt} = \frac{d[C]}{dt} = +k_1(h\nu)\Delta[A] - k_2\,\Delta[B]\,\Delta[C]$$

Where $\Delta[X]$ is the difference between the time-dependent concentration and the equilibrium concentration.

The above, which is a true statement for any chemical reaction (with few trivial changes to adapt the reasoning to other chemical reactions involving more or less species), implies that the kinetics of the reactions are independent of the concentration of the species that affect the optoelectronic properties but only depend on the variation from their equilibrium value.

While $[A]_{eq}$, can be considered constant because it corresponds to the bulk perovskite (variations of the fabrication procedure do not modify its concentration) $[B]_{eq}$ and $[C]_{eq}$ can effectively depend on the procedure used to obtain the perovskite films and can be affected by the crystallite size, the concentration of specific precursors or any other element that has been identified to cause irreproducibility. Nevertheless their values do not enter the equations: this means that, assuming the validity of established chemical knowledge for halide perovskites, the kinetics of self-healing, should not depend on the "state" of the perovskite samples but only on their composition. Our findings can then be extended to samples of $APbI_3$ independently of the fabrication procedure.

Supplementary data:

X-Ray Diffraction Studies

-*a- MAPbI$_3$, MAPI*: As shown in Figure S2, the XRD pattern matches the literature one of pure tetragonal *MAPbI$_3$*.

-*b- High temperature photoactive α-FAPbI$_3$, FAPI*: The XRD pattern (Fig. S3) matches the literature data for FAPI. Except for a peak at 2θ = 12.67° (011) for PbI_2 (space group P -3 m 1 (164)), no extra peaks are seen, notably none for the yellow δ-phase of $FAPbI_3$.

-*c- Low temp. photoactive γ-CsPbI$_3$, CsPI*: As shown in Figs. S4, the XRD pattern matches the literature one, but minor peaks that correspond to the photo-inactive yellow δ- phase (denoted with the asterisk mark) are found as well. The presence of the yellow phase is that for experimental (XRD) reasons, the measurement was done on samples with less encapsulation than those for the FRAP studies, and were in ambient air for the ~1 h of measurements. At the start of the measurement, films were black but at the end of the XRD experiment the edges of the film turned yellowish; this conversion is extremely slow if the film is well encapsulated (as for the FRAP experiments) or stored inside the glove box (> 6 months).

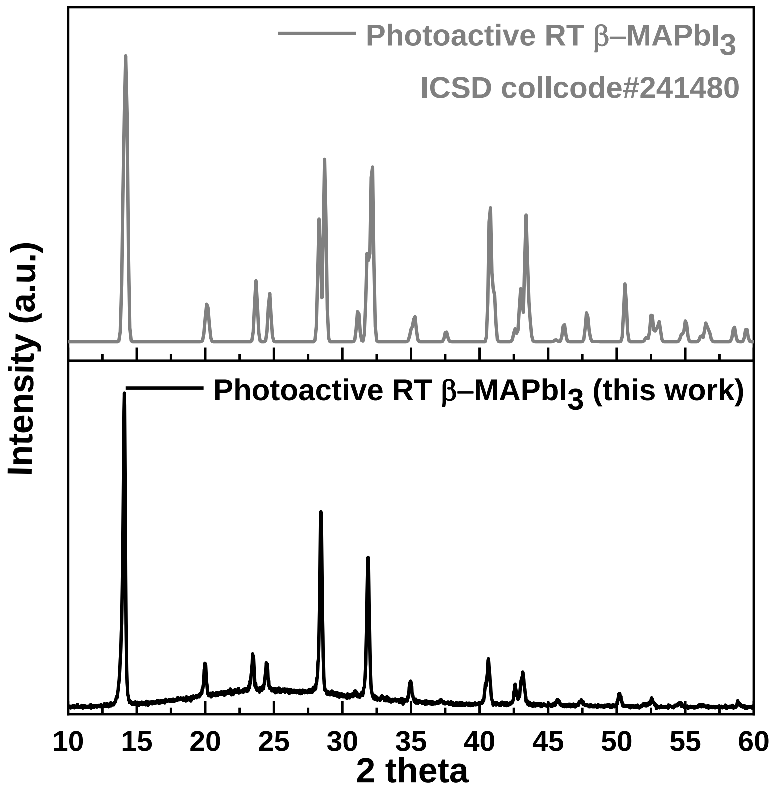

Figure S3. Diffraction pattern of room temperature photoactive β − $MAPbI_3$ phase.

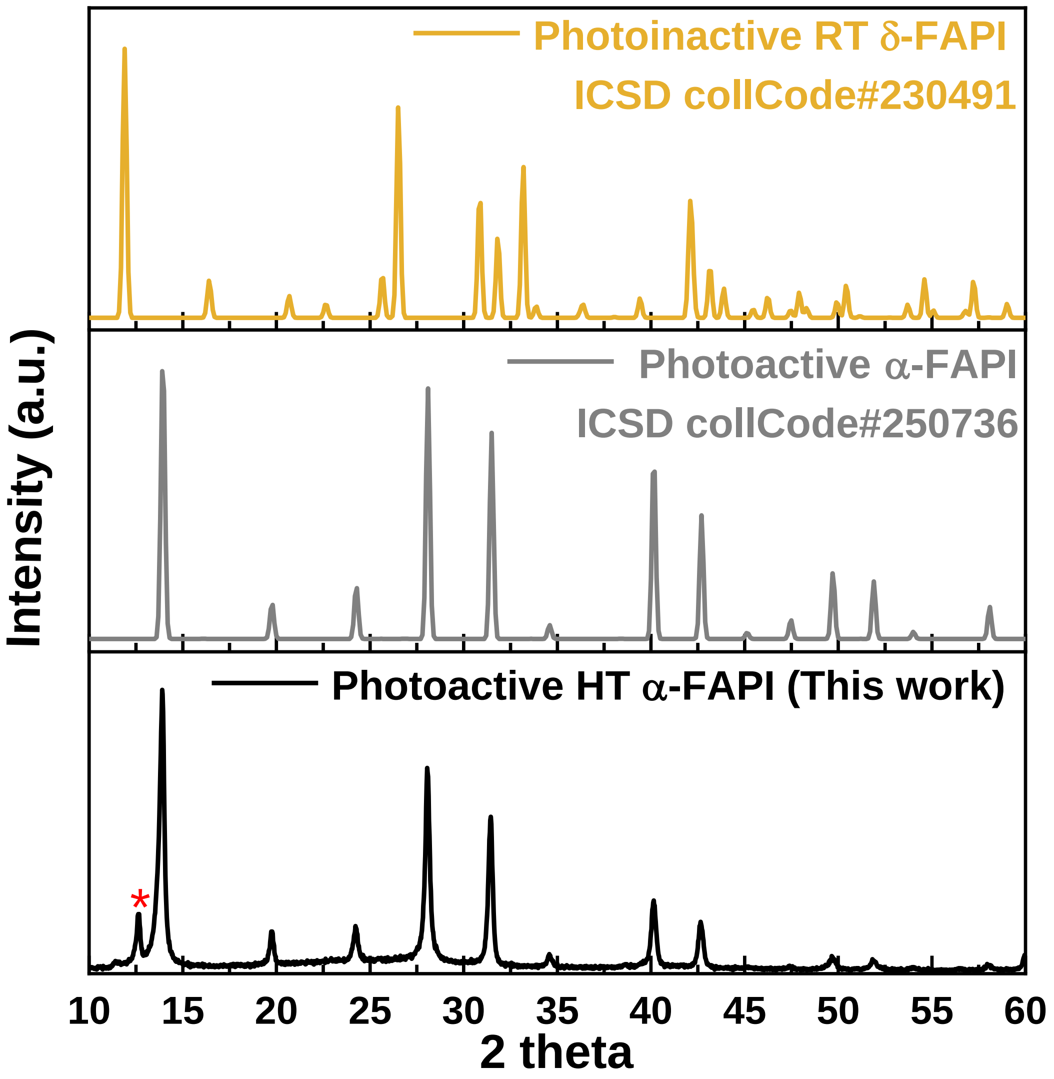

Figure S4. Diffraction pattern of trigonal FAPI (both photoactive black α-phase and photo-inactive yellow δ-phase) along with impurity of PbI_2 indicated with a red asterisk.

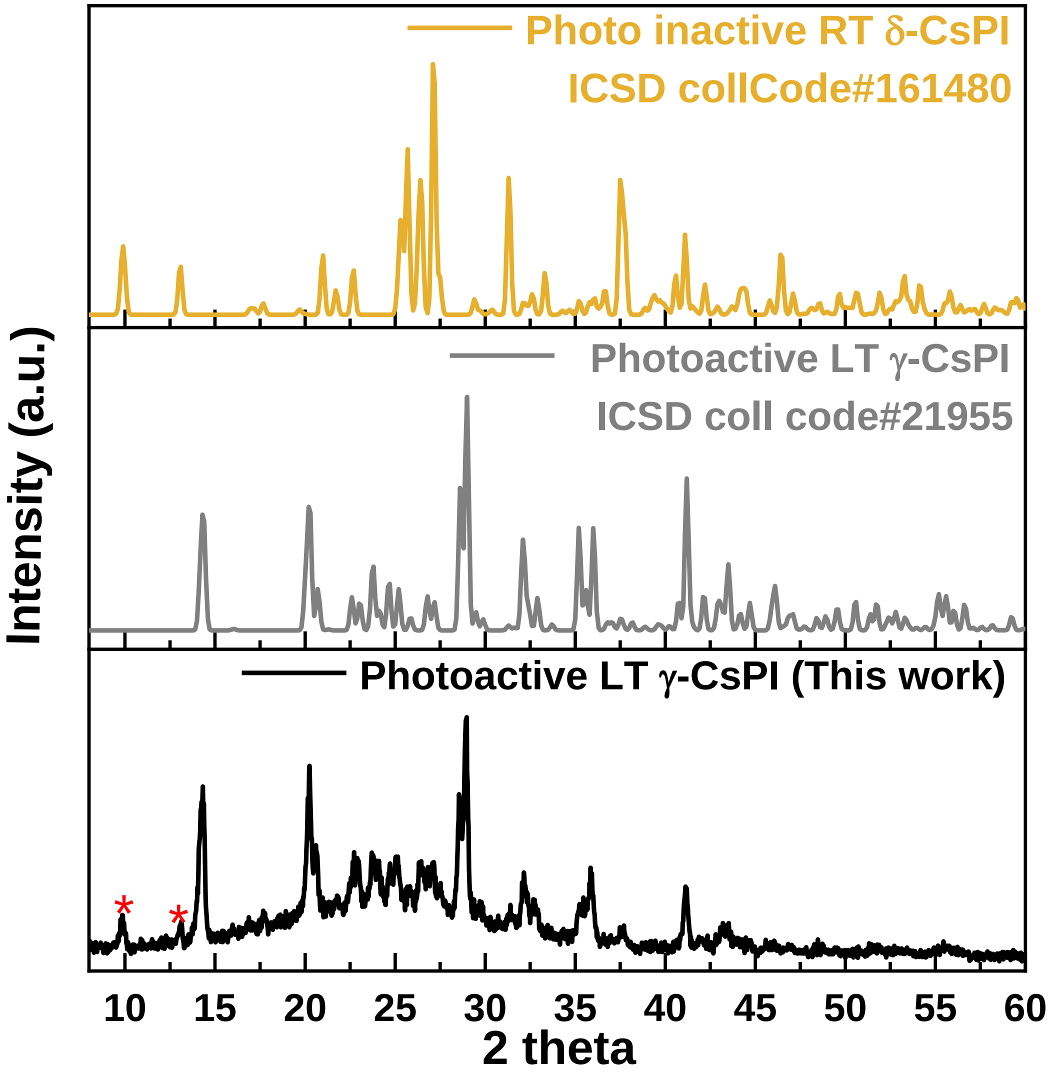

Figure S5. Diffraction pattern of low temperature photoactive $\gamma - CsPbI_3$ phase (major fraction) along with a very small fraction of low temperature photo-inactive orthorhombic $\delta{-}CsPbI_3$ phase, indicated with a red asterisk.

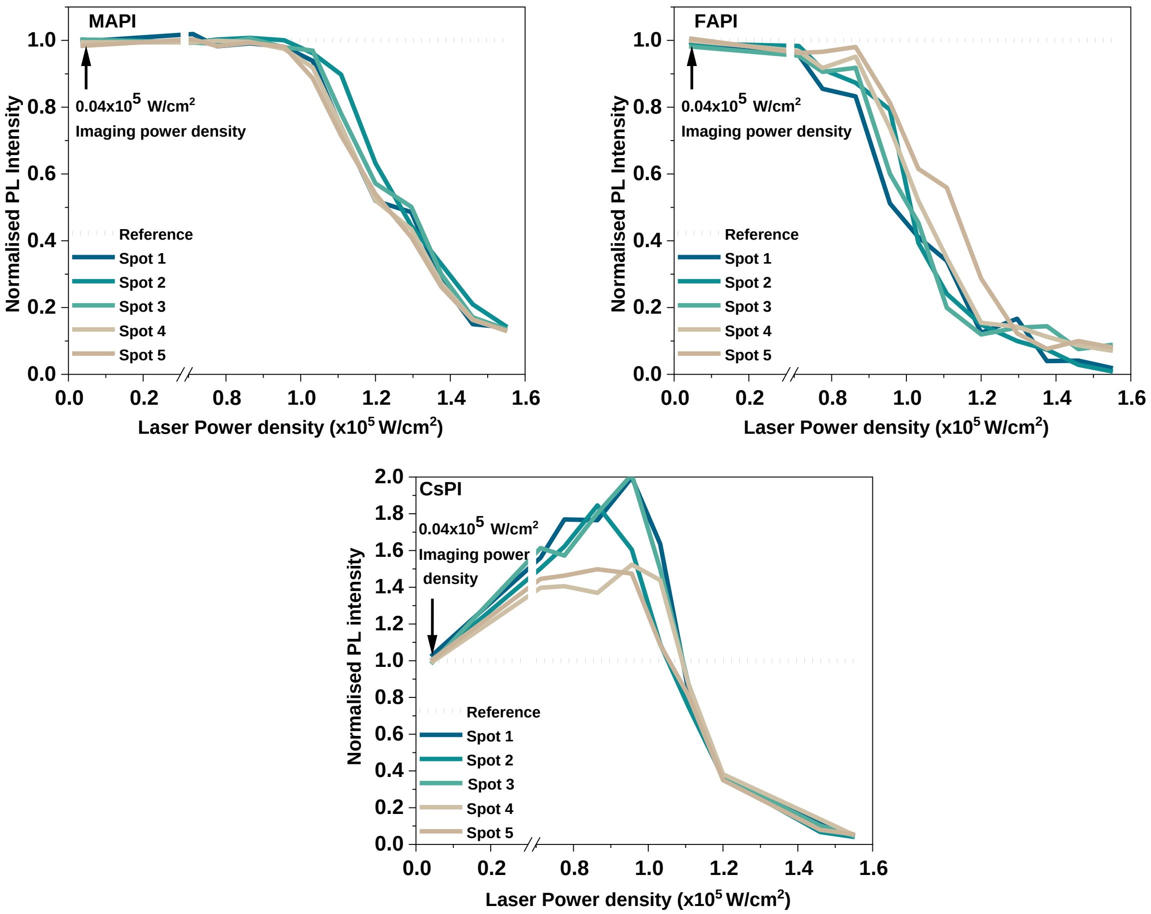

Figure S6. Uniformity in photo-damage threshold and photo-response (photo-damage extent) among encapsulated polycrystalline thin films at varying laser power densities. Spots 1 to 5 represent a single photo-damage event at various spots on the same sample or a different sample of the same perovskite (a) MAPI (b) FAPI and (c) CSPI.

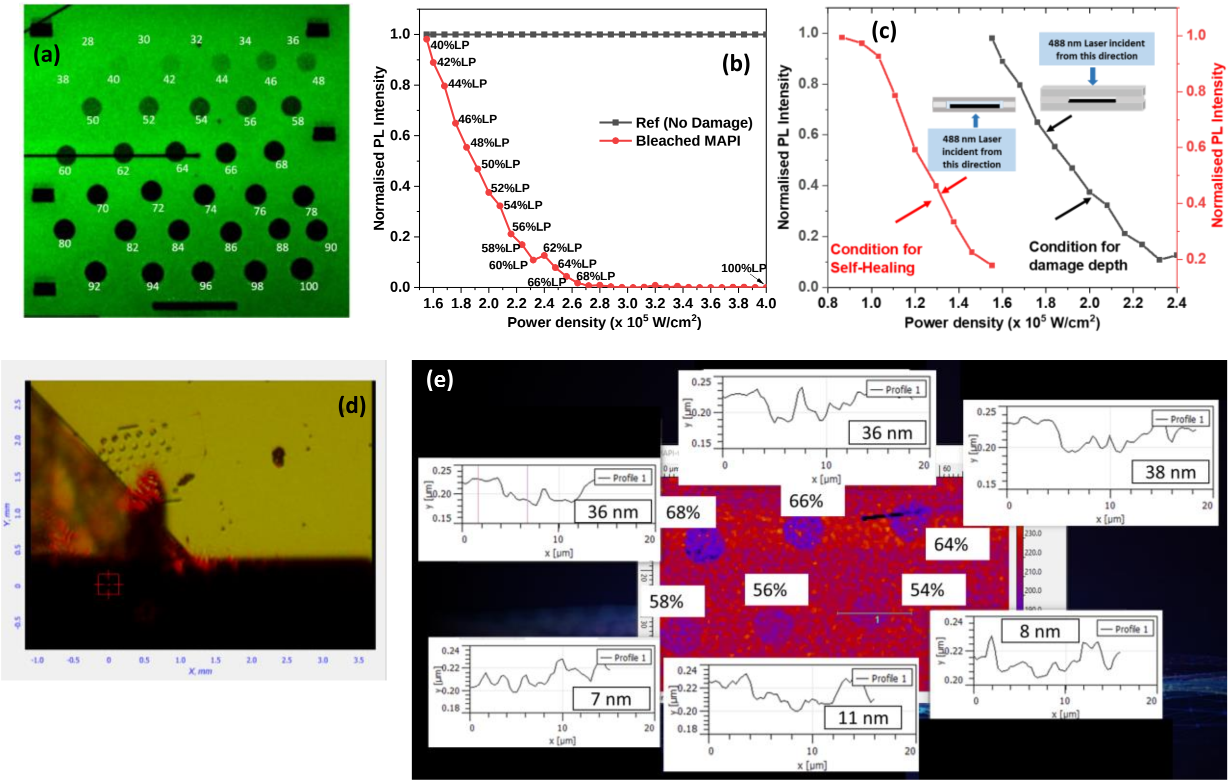

Figure S7. AFM measurement of photo-damaged surface of MAPI film (a) Photo-damage of glass-encapsulated MAPI thin films using varying laser power % of 488 nm denoted with white text below the circular ROIs. (b) Plot of normalized PL intensity vs laser power density (corresponding to the laser power % used in Fig SI 6a), averaged from 5 spots on the sample, where the nearly 68% LP (~ 2.6×10^{5} W/cm^{2}) is enough for 100% PL Loss. (c) Comparison of extent of photo-damage obtained from different power densities used for SH studies and AFM studies, due to the difference in sample architecture. For clarity, the sketch of sample architectures used in either experiment is also presented, where the right architecture is polymer free (d) Image of photo-damaged surface under AFM measurement, and (e) surface roughness measurements to assess material loss: on this image, the files were smoothed and line profiles were performed to measure height. The rms roughness along the lines was quite high relative to the step height, about 10 nm; therefore, we can ignore the depth measured for ROIs exposed to 54-58% LP (~ 2.08-2.24×10^{5} W/cm^{2}), which leads to 80% PL loss. However, ROIs with 90-100% PL loss lead to depths of 36-38 nm, after exposure to 64%-68% LP (~ 2.5×10^{5} W/cm^{2}); so we consider these as representative of pits caused by the intense laser damage.

It is important to note that in the samples for SH measurement, the possibility of pit formation will be reduced due to blocking of the damaged surface with the glass substrate on which the film is deposited (Figure S7c). However structural damage could still occur.

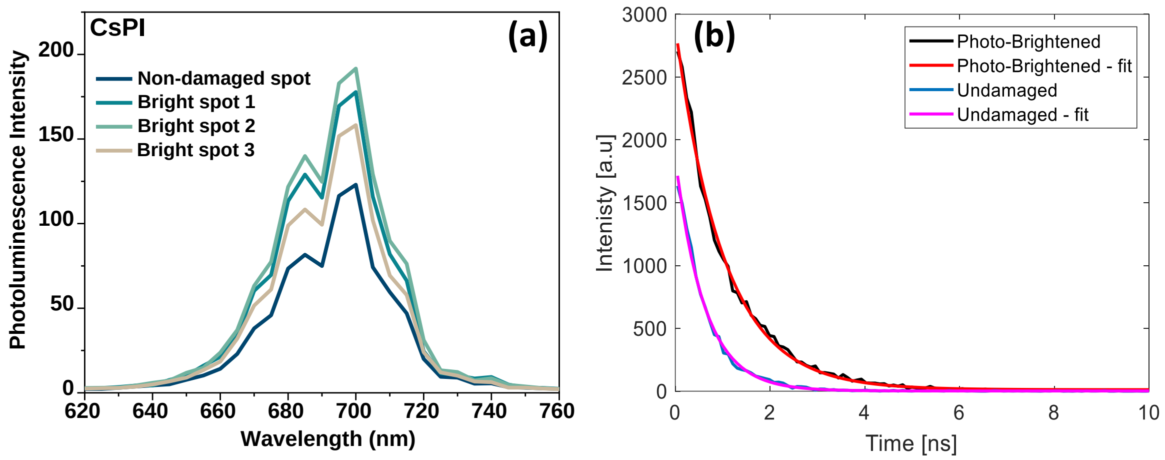

Figure S8: (a) PL spectra of several photo-brightened spots in CsPI film showing increase in PL intensity and no spectral change (spectra were collected from 620 nm to 760 nm with an interval of 5 nm in the confocal system used). (b) Representative time-resolved PL decay curves of photo-brightened (after photodamage) areas and undamaged areas of a CsPI film. A single exponent decay function was fit to each of the curves, yielding $\tau = 1.01$ ns (red) and $\tau = 0.61$ ns (magenta), for the photo-brightened areas and for the undamaged areas, respectively.

We collected the PL lifetime from both the photo-brightened area, which shows a PL increase, and the undamaged area. With a single exponential decay fit from 4 undamaged areas, the average lifetime is 0.62 ± 0.05 ns. A representative TRPL of an unperturbed area and its fitted curve is shown above. From 5 photo-brightened areas, the average lifetime is 0.94 ± 0.07 ns. A representative TRPL of a photo-brightened and its fitted curve are also shown above. The longer lifetime points to improved optoelectronic quality of the film and decrease in the density of non-radiative recombination centers.

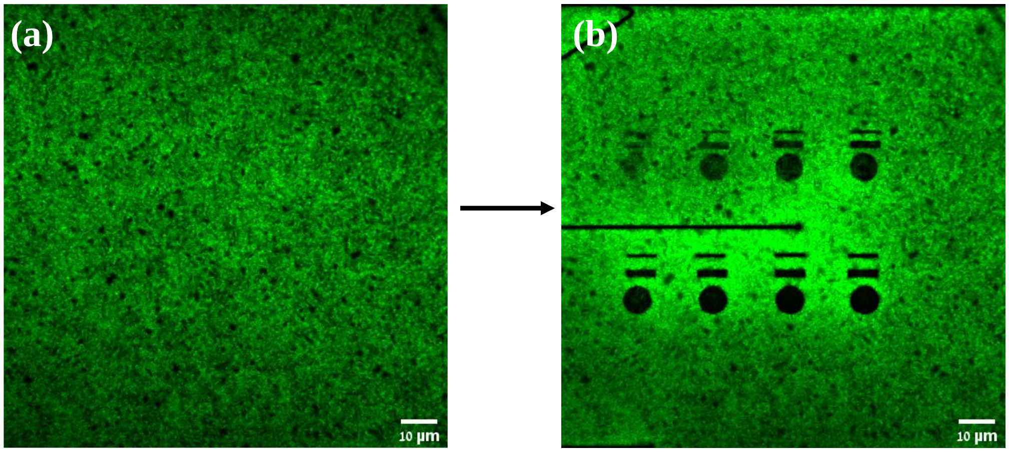

Figure S9: Thin films of photoactive HT α-$FAPbI_3$ (a) Reference image without photo-damage and (b) Image showing photo brightening in the region around ROIs after photo-damage with a series of various power densities.

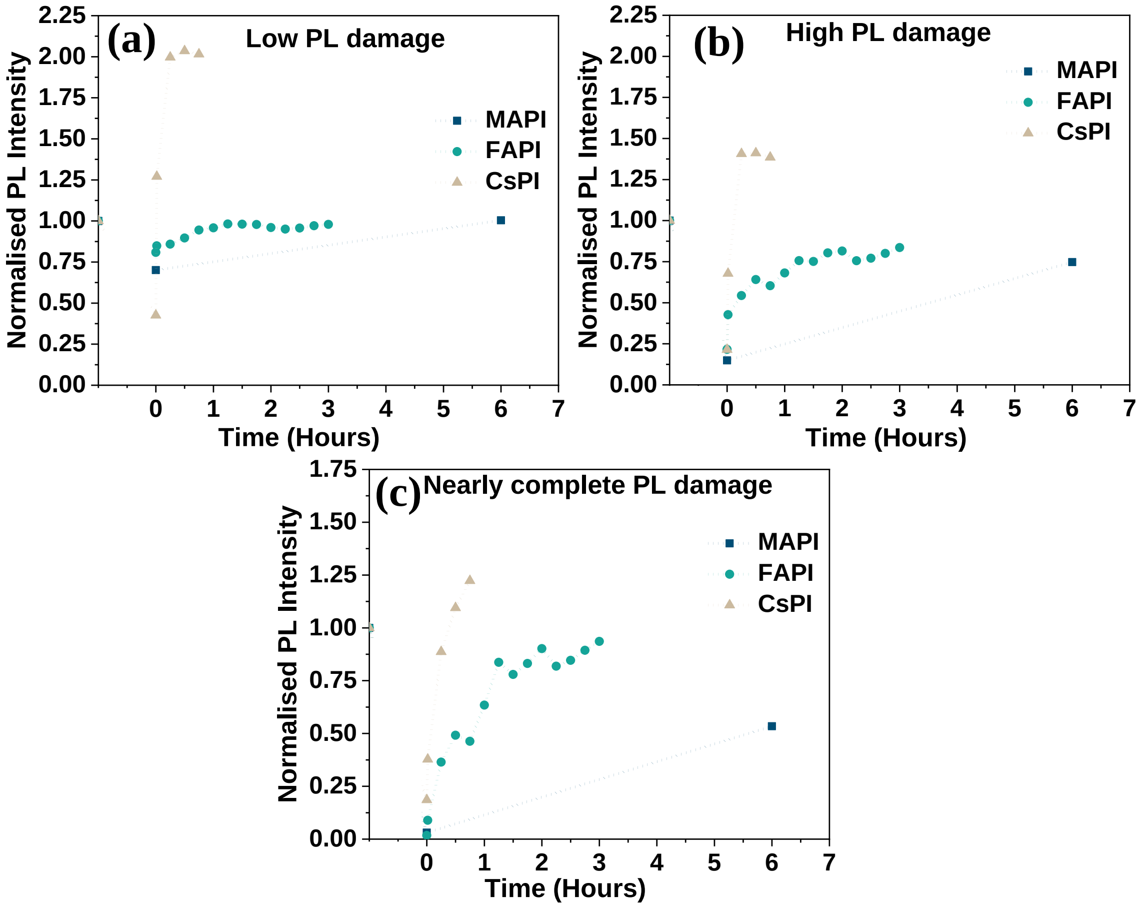

Figure S10: SH kinetics in additional encapsulated polycrystalline films of MAPI (2 data points only), FAPI and CsPI as PL intensity vs. time, using the 488 nm CW laser excitation. Low PL damage (a), high PL damage (b), and nearly complete PL damage (c).

The additional FRAP experiments were performed on an inverted optical microscope setup. Photodamage was performed with a continuous wave blue (405 nm) laser (OBIS) using a 0.5 NA objective onto a diffraction-limited spot (σ ~1.2µm), where the laser irradiated each spot on the film for a few seconds with varying power densities. Following photodamage, the damaged region of the sample was either imaged via a scanning confocal imaging on a single photon avalanche diode, using the same laser at low (undamaging) power, or via wide-field illumination using a blue (405nm) LED (Mightex) and imaged on to an EMCCD detector (Andox Ixon Ultra 897).

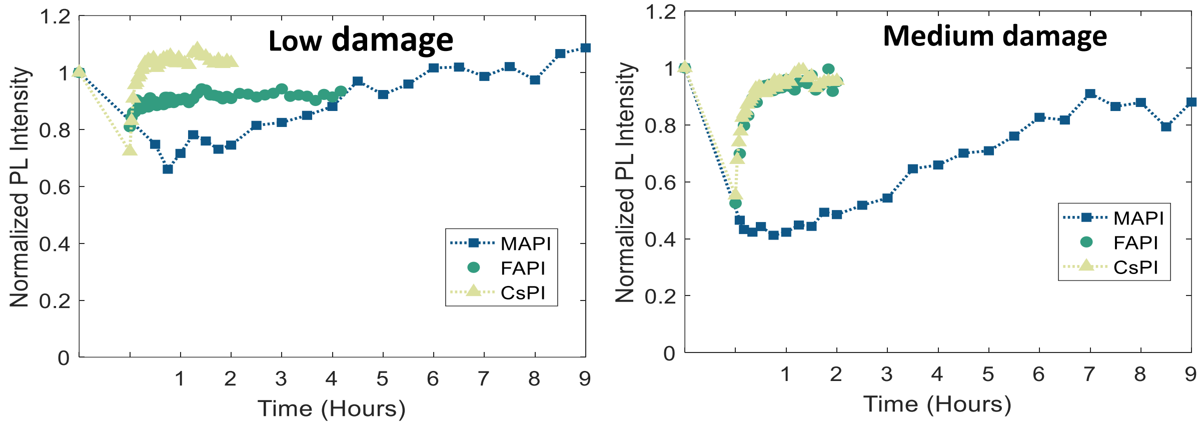

Figure S11: SH kinetics in another set of encapsulated polycrystalline films of MAPI, FAPI and CsPI, using a 405 nm CW laser to damage during several seconds on each spot (no raster scan). The sample was imaged before and after damage using, either scanning confocal imaging (for MAPI and low damage FAPI) or wide field illumination imaging (for CsPI and high damage MAPI). (a) low damage and (b) high damage.

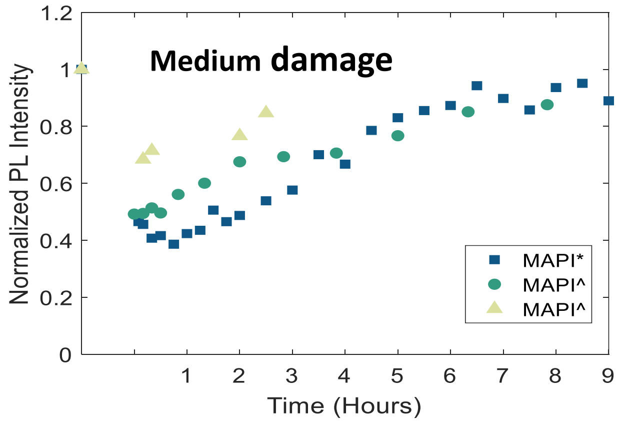

Figure SI2. Two different samples of MAPI (two runs on one of the films) using a 405 nm CW laser to damage during several seconds on each spot (no raster scan). The sample was imaged before and after damage using, scanning confocal imaging. .

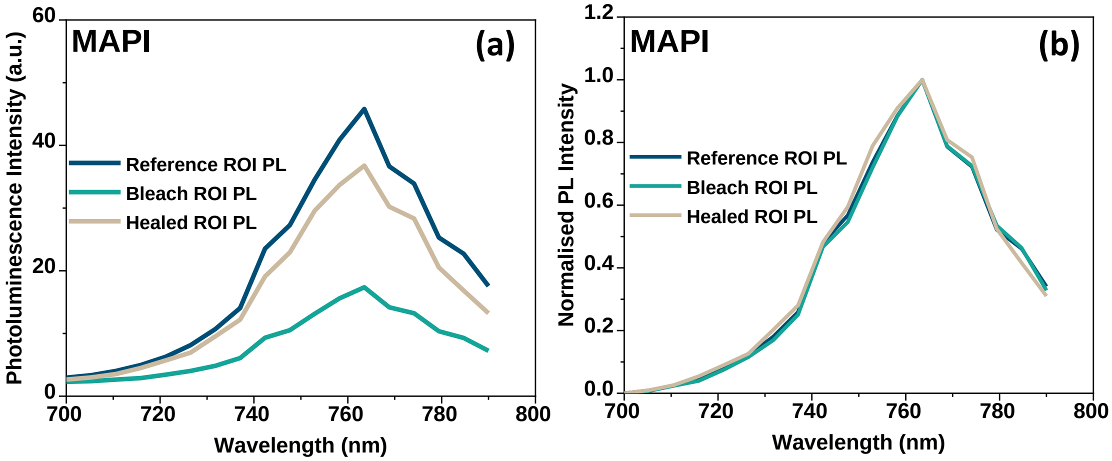

Figure S13: PL spectra of MAPI (a) without normalization, showing healing of ROI and (b) normalized spectra, showing no spectral shift or other change (note the spectra are collected from 700 to 795 nm with and interval of 5 nm in the confocal system used.) The PL spectra of the non-damaged ROI (blue curves), photo-damaged ROI (cyan curves) and the same healed ROI (beige curves) are shown.

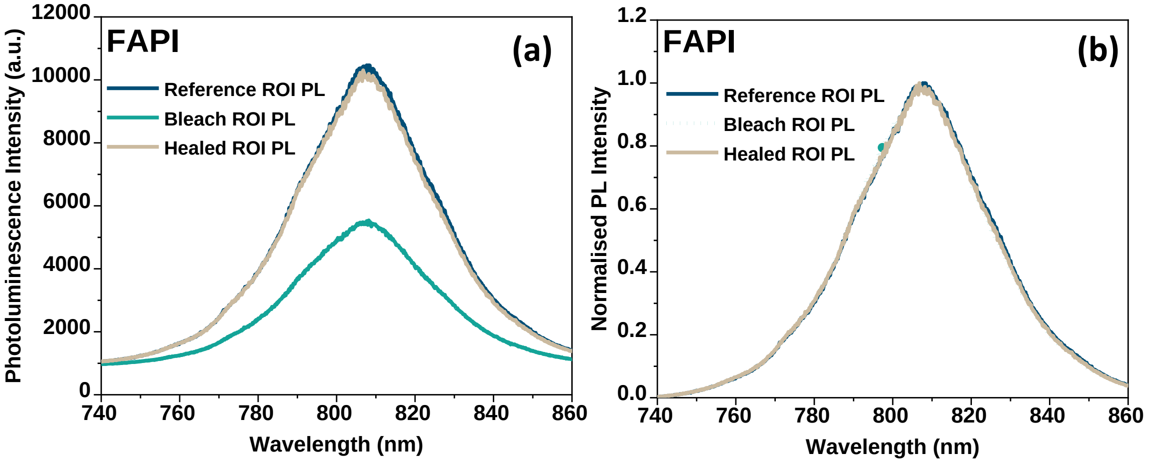

Figure S14: PL spectra of FAPI (a) without normalization, showing healing of ROI and (b) normalized spectra, showing no spectral shift or other changes. The PL spectra of the non-damaged ROI (blue curves), photo-damaged ROI (cyan curves) and the healed ROI (beige curves) are shown. Since the FAPI emission is further into the infrared and outside the detection limit of the confocal system, the spectra were taken *ex situ*, using a spectrometer, which uses a 405 nm laser for excitation.

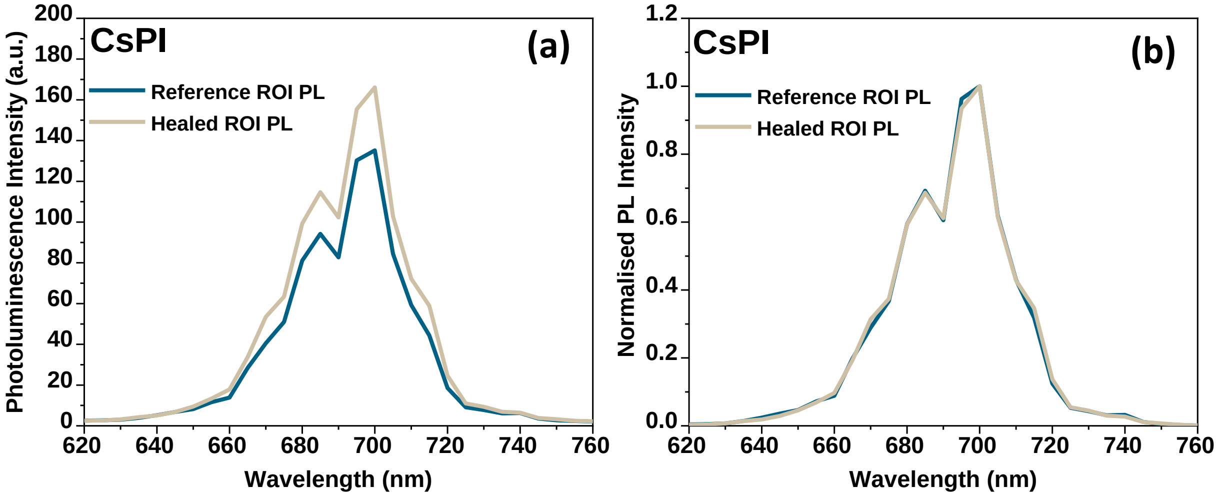

Figure S15: (a) The PL spectra of the non-damaged ROI and the healed ROI showing increase in PL intensity after healing (measurement condition same as fig. S8a) and (b) Normalized PL spectra of the non-damaged ROI and the healed ROI, showing that there is no spectral shift.

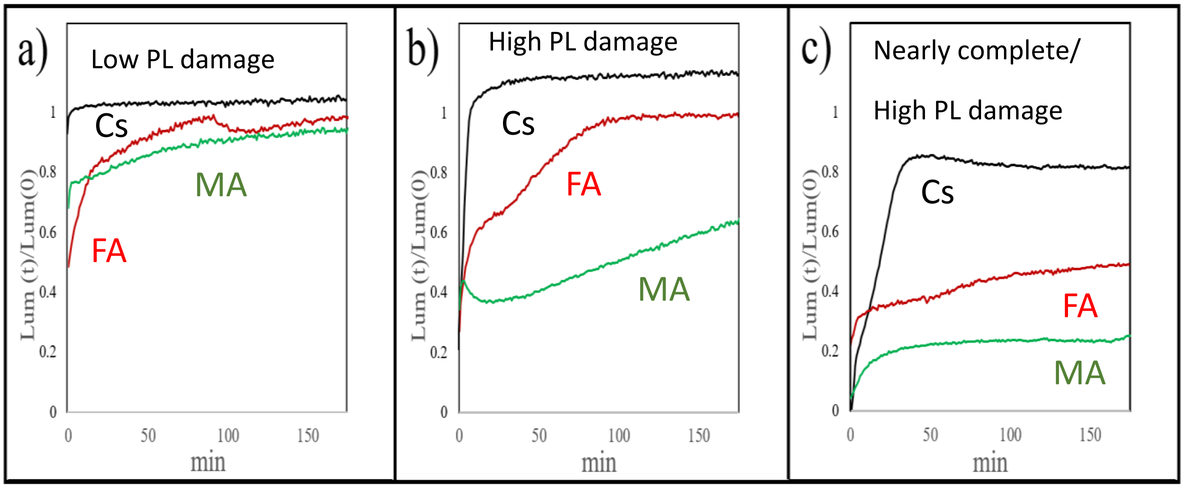

Figure S16: Self-healing kinetics of several damaged ROIs for each of three types of Pb-bromide perovskite single crystals, $CsPbBr_3$, $FAPbBr_3$ and $MAPbBr_3$ (color-coded as shown in the figures) at three different absorbed laser intensities that inflict photodamage, measured as fraction of PL emission before photodamage. The photodamage is **-a-** low, **-b-** high, and **-c-** so high that the PL emission has nearly disappeared after photodamage, as indicated in the figure legends. The experiments were done with a 1-photon confocal microscope, as described in ref. 7. Thus, the near surface region (few hundred nm) was probed. Note that the samples were not encapsulated and were exposed to ambient during measurement. The raw data were taken from the data sets, which are given in ref. 7.

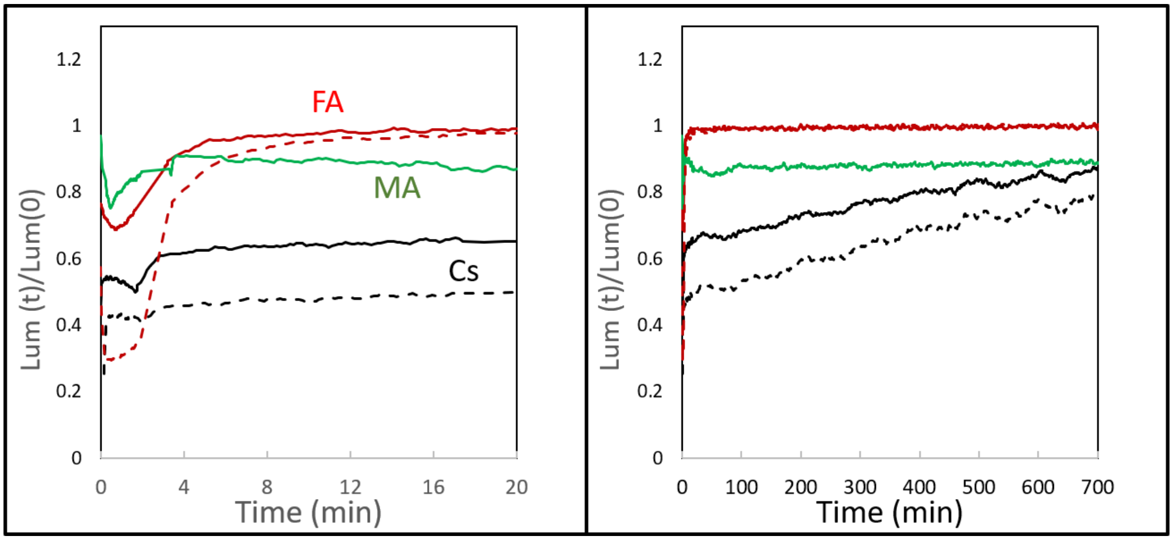

Figure S17: As for Figure SI 16, except that the experiments were done with a 2-photon confocal microscope to cause damage inside the crystals far from the crystal surfaces. *Solid lines*: lower damage; *dashed lin*es: higher damage. LEFT: short times: RIGHT: longer times. No data are shown for $MAPbBr_3$ at high damage levels, as then no PL increase was seen, due to competing photobrightening. For the longer times the higher and lower damage plots of the $FAPbBr_3$ crystals overlap. The raw data were taken from the data sets, which are given in ref. 7.

References:

(1) Chen, Z.; Zhang, H.; Yao, F.; Tao, C.; Fang, G.; Li, G. Room Temperature Formation of Semiconductor Grade α-$FAPbI_3$ Films for Efficient Perovskite Solar Cells. *Cell Reports Physical Science* **2020**, *1* (9), 100205.

(2) Zhao, B.; Jin, S.-F.; Huang, S.; Liu, N.; Ma, J.-Y.; Xue, D.-J.; Han, Q.; Ding, J.; Ge, Q.-Q.; Feng, Y.; Hu, J.-S. Thermodynamically Stable Orthorhombic γ-$CsPbI_3$ Thin Films for High-Performance Photovoltaics. *J. Am. Chem. Soc.* **2018**, *140* (37), 11716–11725.

(3) Ke, W.; Spanopoulos, I.; Stoumpos, C. C.; Kanatzidis, M. G. Myths and Reality of $HPbI_3$ in Halide Perovskite Solar Cells. *Nat Commun* **2018**, *9* (1), 4785.

(4) Kovalsky, A.; Wang, L.; Marek, G. T.; Burda, C.; Dyck, J. S. Thermal Conductivity of $CH_3NH_3PbI_3$ and $CsPbI_3$: Measuring the Effect of the Methylammonium Ion on Phonon Scattering. *J. Phys. Chem. C* **2017**, *121* (6), 3228–3233.

(5) Haeger, T.; Heiderhoff, R.; Riedl, T. Thermal Properties of Metal-Halide Perovskites. *J. Mater. Chem. C* **2020**, *8* (41), 14289–14311.

(6) *Glass | Density, Heat Capacity, Thermal Conductivity*. Material Properties. https://material-properties.org/glass-density-heat-capacity-thermal-conductivity/ (accessed 2022-10-13).

(7) Ceratti, D. R.; Cohen, A. V.; Tenne, R.; Rakita, Y.; Snarski, L.; Jasti, N. P.; Cremonesi, L.; Cohen, R.; Weitman, M.; Rosenhek-Goldian, I.; Kaplan-Ashiri, I.; Bendikov, T.; Kalchenko, V.; Elbaum, M.; Potenza, M. A. C.; Kronik, L.; Hodes, G.; Cahen, D. The Pursuit of Stability in Halide Perovskites: The Monovalent Cation and the Key for Surface and Bulk Self-Healing. *Mater. Horiz.* **2021**, *8* (5), 1570–1586.
